# Quantifying racial inequality in transit access across New York City

**DOI:** 10.1093/pnasnexus/pgag025

**Published:** 2026-05-19

**Authors:** Yunke Zhang, Fengli Xu, Emily Talen, Marta C González, Yong Li

**Affiliations:** Department of Electronic Engineering, Beijing National Research Center for Information Science and Technology (BNRist), Tsinghua University, Beijing 10084, China; Department of Electronic Engineering, Beijing National Research Center for Information Science and Technology (BNRist), Tsinghua University, Beijing 10084, China; Social Sciences Division, University of Chicago, IL 60637, USA; Department of City and Regional Planning and Civil and Environmental Engineering, University of California, Berkeley, CA 94720, USA; Department of Electronic Engineering, Beijing National Research Center for Information Science and Technology (BNRist), Tsinghua University, Beijing 10084, China

**Keywords:** transit accessibility, racial inequality, urban mobility, racial segregation, sustainable transportation

## Abstract

Urban transit networks are crucial for sustainable cities, yet their uneven distribution often results in unequal access to urban resources. Prior studies primarily focused on accessibility within transit networks while overlooking its implications for residents’ behaviors and the historical and political roots of inequality. Using door-to-door transit information and over 66 million mobility records, we examine racial disparities in New York City’s transit accessibility and their impact on mobility behaviors. We find that ethnic minorities have 4.9% lower neighborhood accessibility, 15.7% less job accessibility, and 14.9% poorer access to essential facilities compared to the white population. These disparities exacerbate residential segregation by 26.9% and confine minorities to limited mobility, higher unemployment risks, and longer travel to essential services. Importantly, inequalities persist even after accounting for socioeconomic covariates, housing values, and residential sorting, pointing to the enduring influence of wealth, political power, and discriminatory planning legacies. Simulations indicate that enhancing transit infrastructure in minority block groups could reduce these gaps by up to 49.8%. Our findings highlight the critical need to integrate equity, affordability, and inclusiveness into the design of future sustainable transit systems.

Significance Statement Urban transit systems are fundamental to sustainable development, yet their role in perpetuating racial inequality remains poorly understood. By integrating multimodal transit data with 66 million mobility records, this study reveals that ethnic minorities in New York City face significantly lower accessibility to jobs and essential services. These disparities persist regardless of income or residential sorting. We demonstrate that transit gaps act as a structural mechanism that reinforces residential segregation by nearly 27%. Our findings highlight how contemporary inequality reflects enduring wealth asymmetries and historical discriminatory legacies. Simulations show that targeted infrastructure investments can reduce these gaps by half, providing a critical framework for designing inclusive cities.

## Introduction

Urban transit networks have long played a crucial role in the transition toward environmentally sustainable cities by enhancing energy efficiency and reducing carbon emissions (1, 2). Cities around the world are adopting transit-oriented developments that concentrate resources near transit stations (3, 4). This strategy seeks to offer convenient access to diverse facilities and opportunities through transit networks, encouraging the adoption of sustainable transportation modes (4, 5). Currently, in New York City and Tokyo, over half of the commuters rely on the city’s interconnected transit system for their trips. However, creating an efficient transit network requires substantial investments in well-connected transit infrastructures like buses, subways, and light rails. The uneven distribution of these infrastructures can exacerbate inequality in urban dwellers’ access to essential facilities and opportunities (6–8). According to the United Nations’ estimation, only half of the world’s urban population lives within walking distance of transit stops (9), posing challenges to the equity and inclusiveness goals of cities (10, 11).

Existing research has extensively explored urban residents’ access to resources such as job opportunities and essential facilities (12–14), with significant advances in methodologies for measuring accessibility (12). Early studies used spatial proximity, such as Euclidean distance to the nearest facility (15, 16). Subsequent studies quantified the cumulative number of facilities within a specific spatial range (17, 18), or used a gravity potential measure that incorporates distance-decaying weights (6, 19). Recent intracity research has highlighted the importance of incorporating transit and road networks into accessibility measurements, which assesses residents’ capacity to access facilities through these infrastructure networks (20, 21). These measures, coupled with spatial distributions of subpopulations within cities, have spurred research on the social equity of accessibility, such as the access to job opportunities (6, 22, 23), banks (24), healthcare services (25, 26), parks (17, 27), and schools (28). Notably, there has been a special focus on the inequality in accessibility through transit networks among populations who do not own cars (22, 29).

While these studies show that racial and socioeconomic disparities in transit accessibility are pervasive (20, 22–24, 29), they often stop short of linking access gaps to real-world behavior and segregation outcomes. Most rely on static accessibility measures, without examining how disparities evolve over time or how they can be mitigated. As a result, we still lack a comprehensive framework that connects disparities in transit access to their persistence and change, their consequences for segregation and daily mobility, and their potential remedies through infrastructure investment.

To fill this gap, we organize our study around three research questions: (Q1) What racial disparities exist in transit accessibility across New York City, and how have these disparities changed over the past decade? (Q2) How are these disparities reflected in real-world outcomes, including both experienced segregation and individual mobility behaviors? (Q3) To what extent can targeted infrastructure investments mitigate these disparities? These questions provide a framework that connects measurement, lived consequences, and policy responses into a coherent analysis of transit inequality.

In this article, we leverage large-scale mobility data together with multimodal transit network access to analyze how disparities in accessibility shape urban residents’ behaviors. We organize our framework around three core components: (i) quantifying residents’ transit accessibility to urban facilities and opportunities, (ii) examining how accessibility gaps translate into behavioral consequences, and (iii) evaluating potential strategies to mitigate these inequalities. To address the first component, we calculate a door-to-door travel time matrix within New York City’s multimodal transit system for all block group pairs, and from this derive measures of each block group’s access to jobs and essential facilities (e.g. banks, healthcare, parks, schools) within defined travel-time thresholds. Building on this, we address the second component by combining these accessibility measures with over 66 million aggregate GPS mobility records, enabling us to identify how access inequality corresponds to differences in mobility ranges and burdens. Finally, to address the third component, we simulate planned transit projects that expand services in currently underserved areas, assessing their potential to reduce existing inequalities.

**Fig. 1.**
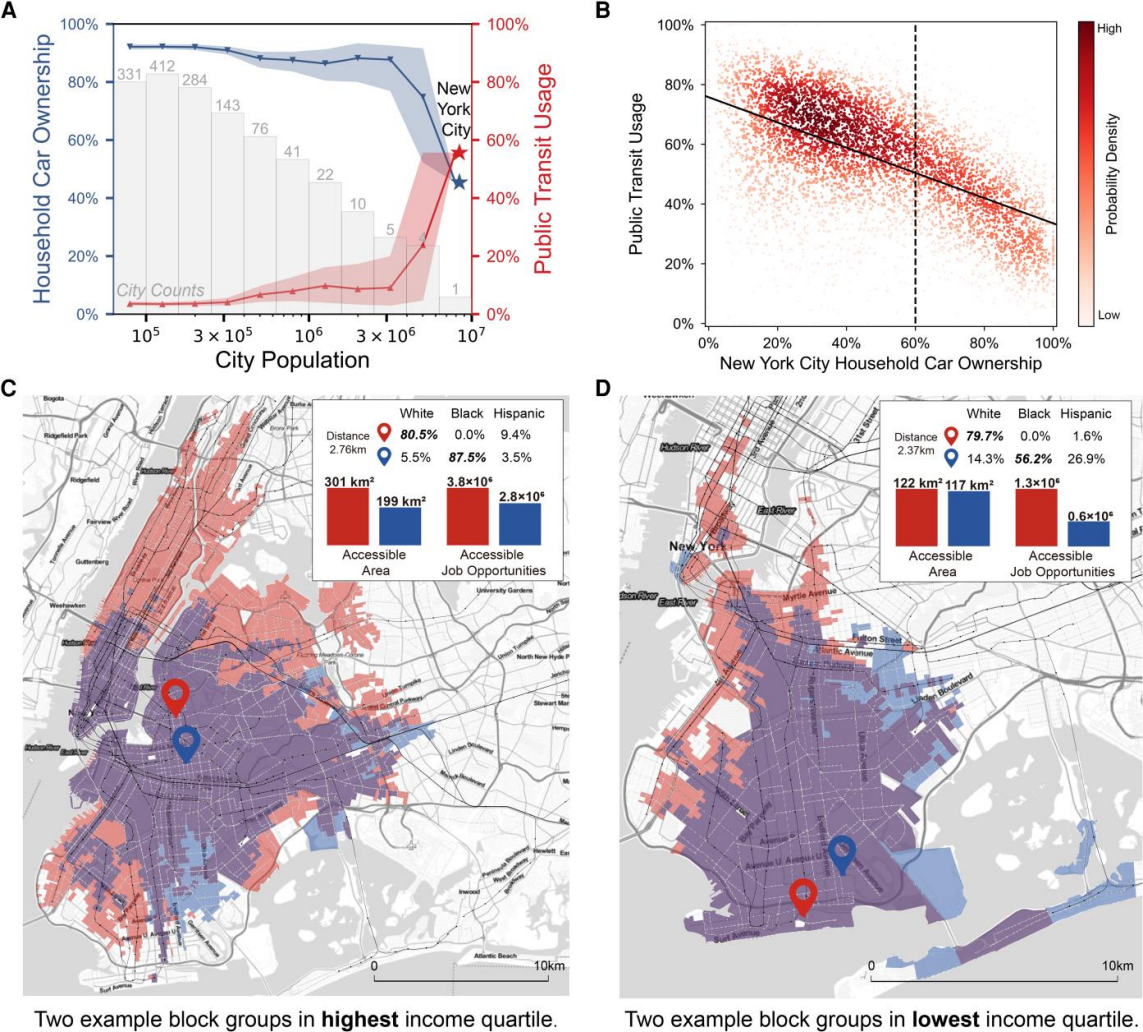
Transit dependence of large cities and disparities in accessibility among block groups. A) The average household car ownership, and average public transit usage of US cities with different population sizes, corresponding 95% CI are shown as shaded areas. Columns show the number of cities in each population bin. B) The correlation between block group’s household car ownership and public transit usage in New York City. The Pearson correlation coefficient is −0.578 (P<0.001). C and D) The accessible block groups in 60 min via the transit network of two pairs of nearby block groups with high- (C) and low- (D) income levels in New York City. Red and blue areas denote block groups that can be accessed by a majority white and a majority black block group within 60 min, respectively. Insets show block groups’ racial composition, area of accessible block groups, and accessible job opportunities. Black and gray lines represent subway/rail and bus routes. Map boundaries are based on NYC Open Data and are used for visualization purposes only.

We focus on New York City, which is the most populous and transit-dependent city in the United States. Almost 60% of commuters utilize its extensive transit network (Fig. 1A), making it a typical case of transit-oriented cities. Moreover, the findings in New York City have important implications for other cities undergoing sustainable transitions. Our research shows pervasive racial inequality in residents’ transit accessibility to essential urban facilities and opportunities. Specifically, black and Hispanic populations have significantly less access to urban block groups, job opportunities, and essential facilities. The relative differences between black and white populations are 4.6, 15.7, and 14.9%, respectively. These disparities persist even after adjusting for socioeconomic factors such as income, car ownership, and transit ridership, and they align with wealth effects, political influence, and discriminatory planning legacies that continue to shape transit geographies today. Moreover, the low transit accessibility confines disadvantaged populations to less racially diverse areas. Current transit network configurations exacerbate residential segregation by up to 26.9% relative to an equitable scenario where all residents had identical accessibility, suggesting a potential mechanism underlying neighborhood isolation in the United States (30). Further, our research shows that transit accessibility is correlated with increased mobility radius, enhanced employment prospects, and reduced travel distances to essential facilities. A mediation analysis shows that racial inequality in transit accessibility contributes between 9.6 and 35.5% of the observed differences in real-world behavior across different racial groups. This result indicates a substantial deprivation of opportunities and heightened living burdens for ethnic minorities due to reduced accessibility. The above inequality motivates our simulations of transit developments in ethnic minority-concentrated areas. Our simulation results suggest that speeding up buses or constructing new subway lines could potentially reduce the accessibility gap by up to 49.8%.

By combining multimodal transit data, census demographics, and large-scale mobility records, our study develops a framework that connects disparities in transit access to their temporal dynamics, lived consequences, and potential remedies. This approach highlights the importance of integrating equity into the planning of sustainable transport systems.

## Results

### Quantifying transit accessibility

Cities vary in their development of transit networks. In the United States, more populated cities usually have lower car ownership and higher public transit dependence (Fig. 1A). Among these cities, New York City stands out as the most transit-dependent, with only 45.4% of households owning private vehicles and over 55.6% of workers commuting by transit. This makes it a unique and important case to analyze the potential inequality in accessibility via its transit system. Furthermore, as public transit increases in New York City, car ownership notably decreases (−0.578,P<0.001) (Fig. 1B), underscoring the critical role of transit networks in cities that aim to promote sustainable transportation modes.

We begin by addressing Q1, quantifying how transit accessibility varies across racial groups in New York City. To quantify transit accessibility of each block group, we use OpenTripPlanner (31), a multimodal trip planning software that measures door-to-door travel time between any two block groups, taking into account all modes of transportation. We then define the block group-level *transit accessibility* as the radius of block group areas and the number of job opportunities or essential facilities (i.e. banks, healthcare facilities, parks, and schools) that can be reached within 60 min using public transportation. Higher values of these accessibility metrics indicate better accessibility (see Methods M1 and Supplementary Note S2 for details). We select two pairs of block groups that are located close to one another and have similar income levels to showcase the large local differences in transit accessibility. In Fig. 1C, despite the spatial closeness and similar high-income levels, the majority white block group (red) has access to the entire Manhattan, while the majority black block group (blue) can only reach the lower Manhattan within 60 min of travel on the transit network. Additionally, the majority white block group has 51% more area of accessible block groups and 36% more job opportunities than the majority black block group. As for two low-income block groups shown in Fig. 1D, we can see that the majority white block group (red) has a slightly wider accessible range than the majority black block group (blue), but its number of accessible job opportunities is more than twice that of the majority black block group, mainly because the subway B and Q lines conveniently link it with lower Manhattan. These large differences in block groups adjacent to each other highlight the role of the transit network in connecting residents with urban resources.

The large disparities in accessibility of predominantly black block groups suggest that they may be deprived of potential opportunities due to unequal transit infrastructures. Moreover, black individuals exhibit a 20% higher transit dependency compared to the white population (Fig. S1). These motivate the need for a comprehensive analysis of the impact of transit accessibility on the behavioral outcomes of block groups.

### Mapping accessibility inequality across urban block groups

**Fig. 2.**
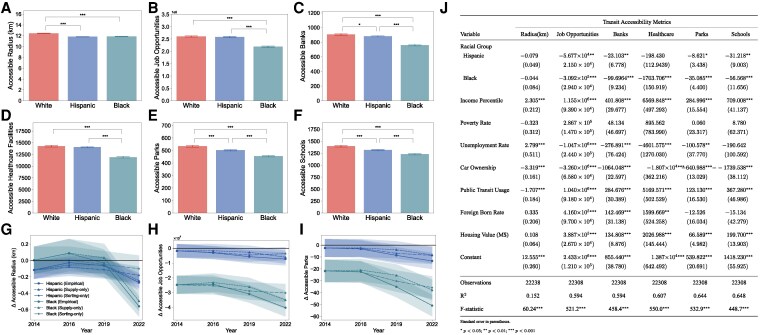
Racial inequality in transit accessibility among block groups. A–F) The average accessible radius (A), job opportunities (B), banks (C), healthcare facilities (D), parks (E), and schools (F) of white, Hispanic, and black populations in 2019. G–I) The temporal trend of racial gaps in accessible radius g), job opportunities (H), and parks I) in 2014, 2016, 2019, and 2022 under empirical, supply-only, and sorting-only scenarios. Shaded areas denote 95% CI. J, Regression coefficients of racial background and socioeconomic variables on accessibility metrics. Significance level: *P<0.05, **P<0.01, ***P<0.001.

We start by examining differences in access across block groups in New York City. Each access metric is averaged over all block groups, weighted by the respective racial populations of each block group. Our results show that there is transit accessibility inequality across different racial groups (Fig. 2A–F). The white population has the highest transit accessibility, while the black population has the lowest transit accessibility, consistently on all metrics. Additionally, differences in transit accessibility across Hispanic and black populations are statistically significant (P<0.001). The white population can travel 12.42 km within 60 min on transit networks (Fig. 2A), while the average travel distances for Hispanic and black populations are 5.03 and 4.60% shorter, respectively. More importantly, Hispanic and black populations can access fewer urban facilities and opportunities for 60-min travel via transit networks. Specifically, the Hispanic population can only access 0.69% fewer job opportunities, 2.78% fewer banks, 1.32% fewer healthcare facilities, 6.09% fewer parks, and 5.77% fewer schools, while the black population can only access 15.74% fewer job opportunities, 16.14% fewer banks, 16.54% fewer healthcare facilities, 15.02% fewer parks, and 11.96% fewer schools than the white population (Fig. 2A–F). We find consistent results when measuring accessibility using gravity-based measures that incorporate a travel time-decay factor to assess the reachable opportunities (12, 32) (see Supplementary Note S2 and Fig. S3).

To rule out confounding effects of socioeconomic status, we fit regression models on transit accessibility, adjusting for potential confounding variables, including income, poverty rate, unemployment rate, car ownership, public transit usage, foreign-born rate, and housing value (Fig. 2J). We take “white” as the reference level, therefore, negative coefficients of Hispanic and black populations reflect their consistent disadvantage to the white populations. Our findings documenting racial inequality across racial groups remain robust after controlling for socioeconomic characteristics. We show that white majority block groups consistently demonstrate higher accessibility compared to Hispanic and black populations, regardless of their socioeconomic status (Fig. S4). Specifically, after we control for the socioeconomic variables, the black population has consistently significant disadvantages in accessing job opportunities and essential facilities. Similarly, the Hispanic population has significant disadvantages in terms of accessible job opportunities, banks, parks, and schools, but no meaningful differences in access to healthcare facilities.

To explore how the observed inequality varies over time, we collect New York City public transit data spanning multiple years (May 2014, 2016, 2019, and 2022). We quantify transit accessibility for each year and show a consistent pattern of racial inequality in transit accessibility metrics throughout this decade (Fig. S5), where the white population have higher access levels than ethnic minorities. This inequality is accompanied by widening gaps between ethnic minorities and white, even after accounting for baseline socioeconomic differences (Figs. 2G–I and S6), complementing previous findings of deteriorated accessibility of black residents (33). For instance, the regression results show that the disadvantage of the black population in accessible parks has increased from 2.40 in 2014 to 13.34 in 2022 (Fig. 2I). Additionally, the gaps in accessible radius, accessible job opportunities and other essential facilities have also consistently increased.

To interpret the sources of widening, we implement two counterfactual series alongside the observed trend (see Methods M1 for details): a supply-only counterfactual that fixes 2014 demographics while allowing the transit network to evolve, and a sorting-only counterfactual that fixes the 2014 network while demographics evolve. Both counterfactuals display increasing racial gaps, indicating that network evolution and residential sorting each contribute to the divergence (Fig. 2G–I and Fig. S6). Specifically, the increases in racial gaps under the supply-only scenarios exceed the increases under sorting-only scenario, suggesting a strong evidence of discrimination in infrastructure construction.

We also probe the historical roots of these patterns by overlaying current accessibility with Home Owners’ Loan Corporation (HOLC) residential security maps from the late 1930s (34, 35). The HOLC city survey graded areas from “A” (best) and “B” (still desirable) to “C” (declining) and “D” (hazardous), reflecting contemporaneous neighborhood risk evaluations shaped by race and poverty and used here as an archival marker of discriminatory appraisal logics rather than a direct blueprint for later FHA redlining (36). Present-day transit accessibility remains strongly stratified along these historic lines. Block groups once graded C/D show significantly lower accessibility across all metrics compared to A/B areas (Fig. S14, P<0.001). This persistence underscores how durable housing and investment regimes, including discriminatory appraisal and public-investment practices, continue to shape the urban transit landscape today.

### Impact of access inequality on experienced segregation and behavior outcomes

Having established disparities in potential accessibility, we next turn to Q2: whether these gaps are reflected in experienced segregation. We combine accessibility measures with residential distributions to estimate how access inequalities translate into spatial segregation patterns.

**Fig. 3.**
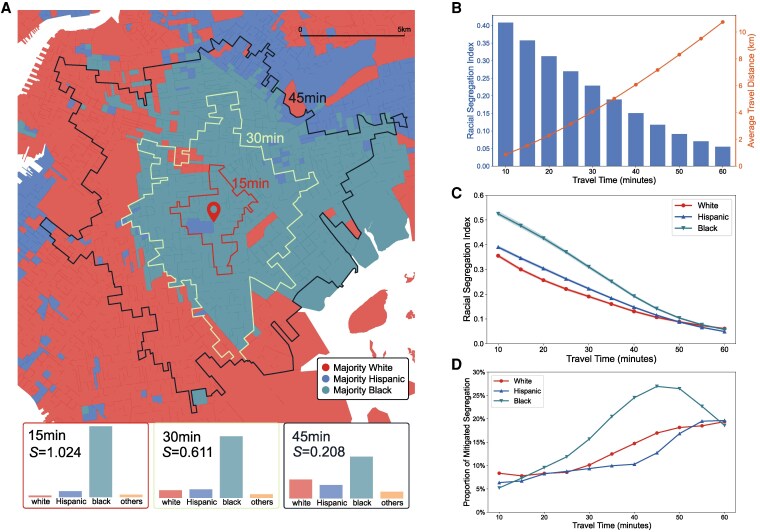
The impact of transit accessibility inequality on experienced segregation. A) Accessible block groups in 15, 30, and 45 min are encircled with bold lines, respectively. Block groups are colored by their majority racial group. Experienced segregation index *S* is calculated as the KL divergence between the racial composition in the accessible block groups and the overall racial composition in the city (see Methods M2 for details). B) The average experienced segregation (columns) and the average travel distance d(t) (line) with 10 to 60 min travel time on the transit network. C) The experienced segregation of white, Hispanic, and black populations with different travel times on the transit network, corresponding 95% CI are shown as shaded areas. D) The proportion of experienced segregation that can be mitigated by equitable transit networks for white, Hispanic, and black populations. Map boundaries are based on NYC Open Data and are used for visualization purposes only.

Efficient accessibility is often seen as a precondition for the availability of wider social opportunities and the convenient utilization of resources (14). A key function of transit networks is linking people living in different areas, promoting social cohesion and generating monetary and intellectual wealth (37, 38). However, the observed unequal distribution of transit accessibility raises concerns that inefficient transit links may hinder some interactions, especially between people of different races. Here, we calculate the experienced segregation *S* of a block group as the divergence between the racial composition in its *empirical accessible block groups* and the baseline composition of the entire city (see Methods M2 for details). As illustrated in the example (Fig. 3A), the 15-min accessible block groups mainly consists of majority black block groups (colored green), generating a higher *S* than 30- and 45-min accessible block groups that cover more diverse block groups. The declining trend of average *S* suggests the transit network can carry residents from segregated block groups to broader and more racially mixed spaces with longer travel time (blue columns in Figs. 3B and S7). However, a comparison of the average *S* across different racial groups (Fig. 3C) shows that the accessible block groups of black and Hispanic populations are significantly more segregated than those of white people under the same transit time.

To measure the impact of accessibility inequality on experienced segregation, we calculate *S* in block groups’ *equitable accessible block groups* under time *t*. These areas are defined as the region within a radius of d(t), the average travel distance under time *t* (orange line in Fig. 3B, see Methods M2 for details). The equitable accessible block groups represents what could be accessed via an equitable and isotropic transit network that disregards differences in racial backgrounds or travel directions. We take the relative difference between *S* in equitable and empirical accessible block groups as the proportion of segregation that can be mitigated by an equitable transit network (Fig. S8). These proportions are positive (Fig. 3D), i.e. the empirical accessible block groups are more segregated than equitable accessible block groups, suggesting the accessibility inequality exacerbates segregation. The black population has the highest proportion of segregation that can be mitigated, up to 26.9% at the travel time of 45 min, followed by white and Hispanic populations. Our findings suggest that the current transit system is confining majority black block groups to segregated areas instead of connecting them with more diverse races. Surprisingly, despite better transit accessibility, the white population is 7% to 43% more impacted by the inequitable transit network than the Hispanic at a travel time of 30 to 50 min (Fig. 3D). These findings demonstrate accessibility inequality as an important mechanism of neighborhood segregation.

**Fig. 4.**
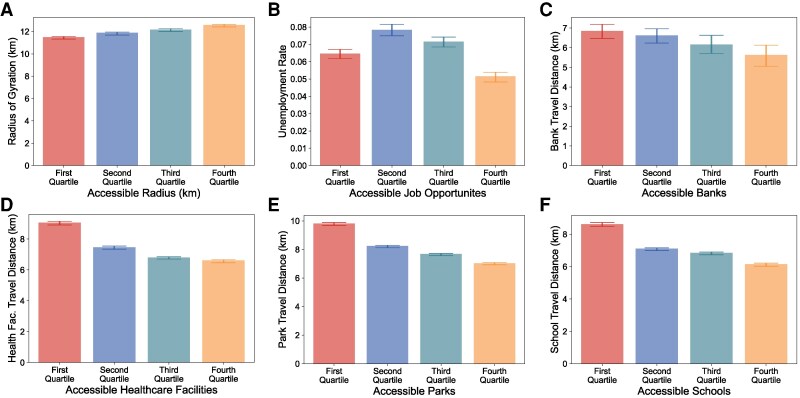
Linking transit accessibility inequality with discrepancies in real-world behaviors. A–F) The average radius of gyration (A), unemployment rate (B), average travel distance to banks (C), healthcare facilities (D), parks (E), schools (F), and corresponding 95% CI of block groups in each accessibility quartile.

Beyond segregation, we also examine how transit accessibility shapes daily mobility behaviors (39). Specifically, we use mobility data obtained from Safegraph (40) which records aggregated mobility counts from block groups to facilities. We compute behavioral indicators of block groups, including the radius of gyration and average travel distances to visit essential facilities like banks, healthcare facilities, parks, and schools (see Methods M3 and Supplementary Note S3 for detailed descriptions). Our findings suggest a clear association between transit accessibility inequality and real-world behavior disparities (Fig. 4). Block groups with a larger accessible radius by transit network have notably broader mobility radius (Fig. 4A). The radius of gyration of block groups with the highest accessible radius quartile is 9.5% higher than that of the lowest quartile group. Moreover, we observe a negative correlation between the average travel distance taken to visit essential facilities and the number of facilities accessible to that block group (Fig. 4C–F, P<0.001). This implies that low transit accessibility can result in fewer essential facility choices, which may compel residents to have a higher travel burden to meet their basic financial, sanitary, leisure, and educational needs. When comparing real-world behaviors across block groups of different racial backgrounds, we find that differences in behavior are also associated with racial background. In particular, Hispanic and black populations have consistently lower radius of gyration, higher unemployment rates, and longer travel distances to visit essential facilities, which are consistent with their disadvantages in transit accessibility (Fig. S10). Notably, compared to the white population, black people have a 13.5% smaller radius of gyration, 82.1% higher unemployment rate, and 1.0, 5.2, 8.4, 7.3% longer travel distance to banks, health facilities, parks, and schools.

**Fig. 5.**
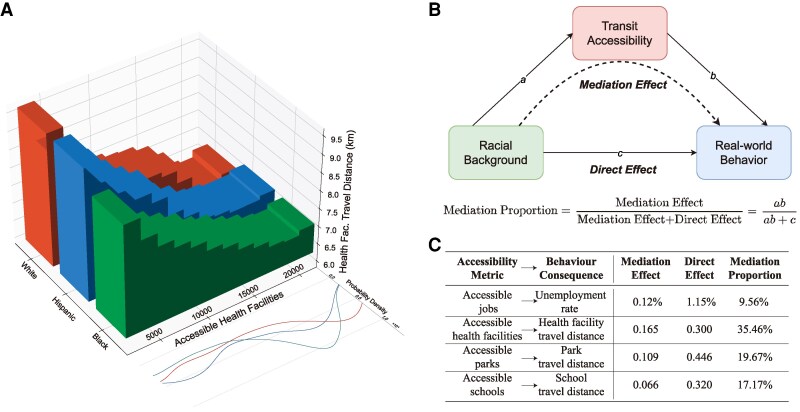
Associations between transit accessibility inequality and racial disparities in mobility behaviors. A) Travel distance to health facilities across populations of different races with different health facilities accessibility. The probability density functions of each race’s accessible health facilities are plotted as the curves beside. B) Illustration of the mediation decomposition. We use this framework to partition the observed disparities in mobility behaviors into a component directly associated with race and a component statistically associated with transit accessibility (41). The mediation proportion is defined as the share of the accessibility-associated component relative to the total association (42). C) Statistically significant mediation proportions of transit accessibility metrics in the observed racial disparities of corresponding mobility outcomes (two-sided *t*-test, P<0.01).

We further investigate how differences in transit accessibility across block groups with different racial backgrounds are associated with mobility outcomes, using a mediation-style decomposition of associations. For example, the travel distance to health facilities is negatively correlated with the number of accessible health facilities (Fig. 5A). Black and Hispanic populations tend to have lower levels of accessible health facilities, which is statistically associated with longer travel distances to reach them. To quantify this, we apply a mediation framework (41) to decompose the observed disparities in mobility outcomes into a direct association with race and an indirect association operating through transit accessibility (42) (Fig. 5B, see Methods M3 and Supplementary Note S3 for details). Our analysis shows that the differences in job accessibility account for 9.56% of the observed disparity in unemployment rates, and differences in access to healthcare facilities, parks, and schools are associated with 35.46, 19.67, and 17.17% of the observed disparities in travel distances to these facilities, respectively (Fig. 5C, see Table S6 for regression coefficients). These results highlight that disparities in transit accessibility are strongly associated with disparities in mobility behaviors, demonstrating the need to assess and mitigate the real-world representations of inequalities in established transit systems.

### Exploring policies for mitigating accessibility inequality

Finally, to address Q3, we evaluate potential remedies. The racial inequality we observe in New York City could be have several underlying reasons, including the tendency of high-income white residents to move to areas with better accessibility (43) and insufficient investment in certain areas. In order to investigate the role of infrastructure distribution in mitigating accessibility inequality, we focus on public housing districts in New York City, where residents have less economic capacity to relocate to other regions (44). Public housing developments are publicly funded housing provided by the New York City Housing Authority (NYCHA). These are meant to provide affordable housing for more than 400,000 low- and moderate-income residents (45) and to their access to public and community services. However, recent studies have shown that NYCHA housings have dilapidated living conditions, high incarceration rates, and health risks (46, 47). As a result, stakeholders are seeking to make public housing districts more adequately invested and inclusive (48, 49), by advocating for transit-oriented development, which involves integrating the development of affordable housing and accessible transit services (48).

**Fig. 6.**
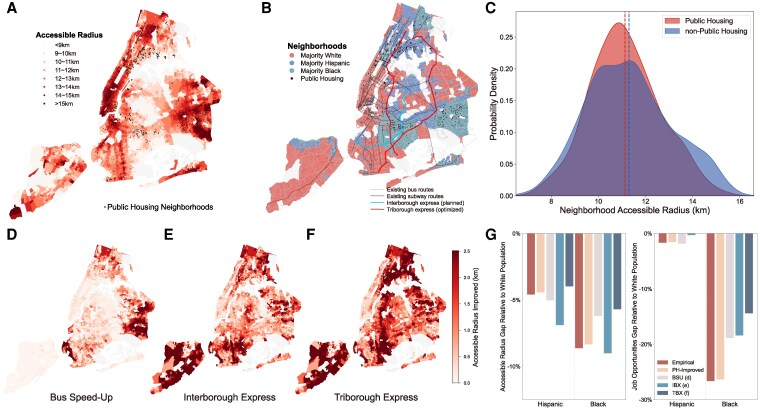
Mitigating accessibility inequality by improving transit infrastructures in public housing districts. A) Spatial distribution of block group accessible radius and public housing developments among block groups in New York City. Colors represent the block group’s accessible radius. Block groups with public housing developments are marked with black dots. B) Spatial distributions of the majority racial group within groups, along with the current and proposed transit infrastructure. C) Probability density plots of accessible radius for public housing block groups and nonpublic housing block groups. Dashed lines represent average values. D–F) Block groups’ improved accessible radius under bus speed-up (D), interborough express (E), and triborough express (F) simulation scenarios. G) Transit accessibility gaps of Hispanic and black populations relative to the white population in the empirical and four simulation scenarios. Map boundaries are based on NYC Open Data and are used for visualization purposes only.

We compare the transit accessibility between block groups with public housing (public housing block groups) and their counterparts (nonpublic housing block groups). We find that public housing is concentrated in block groups with the lowest accessible radius (lighter areas in Fig. 6A), with the majority of these block groups being predominantly Hispanic or black (Fig. 6B). In 2022, the average accessible radius of public housing block groups is 11.13 km, which is significantly lower than the average accessible radius of nonpublic housing block groups (11.30 km, P<0.01, see Fig. 6C). This significant difference shows the disadvantage of public housing block groups in transit accessibility, which creates additional burdens for disadvantaged residents. As a second exercise, we explore the feasibility of mitigating citywide racial accessibility inequality by improving the transit infrastructure in public housing block groups. We first simulate a public housing-improved (PH-improved) scenario where public housing block groups have the same level of transit accessibility as the nonpublic housing block groups, i.e. the accessible radius of each public housing block group is increased by 0.18 km to fill the gap in Fig. 6C. Under this scenario, Hispanic and black populations’ relative accessibility gaps to white people can be reduced by 3.5% on average (Fig. 6G).

The above simulation shows transit accessibility inequality could be relieved by providing better services to minority-concentrated regions. Here, we provide a more comprehensive simulation analysis of three scenarios aligning with real-world development plans and established transit expansion models (Fig. S13). Following the New York City Department of Transportation’s Better Buses Action Plan (50), we design a bus speed-up (BSU) scenario involving a 10% speed increase for bus services in three boroughs with high concentrations of public housing and ethnic minorities. Another scenario involves the planned opening of the Interborough Express (IBX) (51), a light rail project connecting currently deprived regions in Brooklyn and Queens (the blue line in Fig. 6B). Finally, we apply a well-known mathematical programming algorithm designed for transit network extension (52) on the existing subway system (see Supplementary Note S4 for details), which generates two optimized subway lines (Triborough Express, TBX) linking public housing-concentrated areas in three boroughs (red lines in Fig. 6B). Under these simulation scenarios, transit accessibility increases by 0.52 (BSU), 1.18 (IBX), and 1.66 (TBX) km on average, with a more pronounced improvement in peripheral ethnic minority block groups (Fig. 6D–F).

The consequences of accessibility gaps between ethnic minorities and the White population under these scenarios exhibit distinct patterns. Under official plans such as the BSU and IBX projects, the gap in accessible radius between minority and white populations slightly increases. By contrast, racial gaps in accessible job opportunities and essential facilities decline consistently across all interventions (Figs. 6G and S12). On average, job and facility accessibility disparities are reduced by 5.1% under BSU, 22.8% under IBX, and 49.8% under TBX. These results highlight the potential for targeted, transit-oriented development strategies to substantially mitigate existing inequalities. At the same time, they underscore the importance of considering complementary measures to prevent unintended consequences, such as gentrification and displacement, that may offset equity gains.

## Discussion

The Sustainable Development Goals envisioned by the United Nations (SDG 11.2 “Affordable and sustainable transport systems”) emphasize the significance of expanding urban transit with special attention to the needs of vulnerable groups. Our study is particularly relevant as the United States recently introduced a trillion-dollar Bipartisan Infrastructure Law (53) to invest in new transit infrastructure and put a special emphasis on sustainable development. Building inclusive sustainable transit networks is an urgent need for the future development of cities.

This study presents a comprehensive understanding of accessibility inequality embedded in the transit network of New York City. We construct an analytic framework linking urban block groups’ transit accessibility, demographic backgrounds, and mobility behaviors. Here, we extend the understanding of accessibility inequality to link it with disparities in real-world behaviors. We find that areas with limited accessibility experience restricted activity spaces, heightened risks of unemployment, and increased travel burdens to essential facilities. These findings suggest the importance of underscoring the urban transit network’s role as both the driver and key solution to critical social problems such as time poverty (54) and concentrated unemployment (55). Future research should also investigate the impact of public transit accessibility on more general outcomes, such as overall health and quality of life.

Facilitating social mixing in urban space is a fundamental principle in the development of sustainable cities and communities. Following Schelling’s classic segregation model (56), extensive research is devoted to measuring residential and experienced segregation based on race, income, and other factors, along with its detrimental effects (30, 38, 57). Through an analysis of transit accessibility, this study establishes a connection between segregation and the uneven distribution of urban transit infrastructure, highlighting how inequitable transit access can worsen urban residential segregation by confining disadvantaged populations to more segregated areas. This reinforcement of segregation could be associated with historical discriminatory practices, such as redlining. To mitigate these adverse effects, future planning for sustainable transit networks should prioritize social inclusiveness as a core guiding principle, ensuring equitable transit connections between urban opportunities and marginalized block groups.

The gaps in transit accessibility between ethnic minorities and the white population have widened since 2014 (Fig. S5). This trend persists under both the supply-only and sorting-only counterfactuals (Figs. 2 and S6), showing that network evolution and residential sorting each contribute to divergence. We conduct preliminary simulations of targeted service improvements in under-served areas, which suggest potential to narrow disparities. These exercises, however, are partial-equilibrium and hold housing prices and residential location fixed. In practice, new transit investment is often capitalized into higher rents and can trigger further sorting, so equity gains may be offset—or even reversed—without complementary housing measures (58, 59).

Consistent with this mechanism, our regression models show that block groups with higher housing values exhibit systematically greater potential transit accessibility across all metrics, even after adjusting for income and other socioeconomic characteristics (Fig. 2J). This pattern reflects both wealth-based sorting and the capitalization of transit quality into property values, as emphasized in the classic literature on urban land markets (60, 61). In effect, higher-wealth areas are better able to secure and retain high-quality transit services, while minority-concentrated block groups—where housing values tend to be lower—face structural disadvantages in access even when incomes are comparable. This aligns with recent evidence that transit improvements disproportionately benefit higher-income neighborhoods through capitalization effects (62). Moreover, accessibility remains significantly lower today in neighborhoods that were graded poorly in the HOLC security maps (Fig. S14), underscoring the persistence of political-economy and discriminatory siting legacies (34, 35).

Beyond wealth and historical discrimination, political influence has long shaped the geography of urban infrastructure. Classic urban political economy research emphasizes that infrastructure decisions often reflect the preferences of politically powerful groups rather than neutral planning logics (63–65). While our study cannot directly measure representation or lobbying power, the persistence of disparities after controlling for income, housing values, and sorting is consistent with the hypothesis that unequal political voice has historically guided where high-quality transit was built and maintained. This highlights that equity in accessibility cannot be achieved solely through technical planning but also requires institutional reforms to ensure marginalized communities have stronger influence in transit decision-making.

This interpretation connects to a broader political-economy perspective: the uneven geography of transit access reflects not only contemporary sorting but also long-standing asymmetries in wealth, political influence, and institutionalized discrimination (66, 67). It reinforces the need to integrate transit planning with affordable housing and antidisplacement policies (48, 49). Directing more equitable funding to minority-concentrated neighborhoods could ease time and financial burdens, but safeguards are required to prevent displacement and capture by higher-income in-movers, as seen in cases of “green gentrification” (68). A more durable strategy couples service and reliability upgrades with affordability measures such as income-restricted housing near stations, inclusionary zoning, community land trusts, and fare equity programs. Although implementation faces political and fiscal obstacles, including past reductions in MTA funding (69) and local opposition to line extensions, our findings provide a strong rationale for integrated transit–housing strategies to achieve equity goals.

Our study has several limitations. We perform large-scale quantitative measurements of transit accessibility, whereas qualitative individual experiences engaged with various stakeholders were not explicitly considered. While we leverage regression and causal mediation analyses to address potential confounding effects, gaining a deeper understanding of precise causal mechanisms necessitates supplementary causal methodologies, such as instrumental variable analysis or panel data analysis. In addition, this study uses public datasets, which may have limited accuracy and timeliness in rapidly changing urban environments. To mitigate this, we employ up-to-date census and mobility data, which have been demonstrated to offer high coverage and small sampling biases (70, 71). Moreover, our accessibility measures capture direct, single-destination trips. In reality, many travelers combine stops through “trip chaining,” which can lower effective costs in peripheral areas or add scheduling burdens in central ones. Although we cannot observe such patterns with current data, prior work shows that trip chaining can substantially reshape measured mobility outcomes (72). Future research could use higher-frequency activity data to incorporate this dimension. Finally, our study focuses on New York City, limiting the generalizability of our findings to other cities. Because our analysis is bounded to destinations within municipal NYC, central areas are mechanically advantaged while peripheral block groups may appear more isolated than they are in practice, as some residents access opportunities in adjacent counties. Nonetheless, the current state of New York City serves as a reference for the future sustainable development transition in other cities. Future works should also adopt broader metropolitan definitions and compare across global cities to better understand how accessibility inequality varies across different urban settings.

## Materials and methods

### M1. Quantifying transit accessibility

To quantify block group-level transit accessibility, we first derive the door-to-door travel time by public transit between each pair of block groups using OpenTripPlanner (OTP) (31). OTP is a routing engine that builds a multimodal transit network system from General Transit Feed Specification (GTFS) datasets (73) and OpenStreetMap (OSM) road network data (74). We first build an “OTP instance” by creating an OTP graph using New York City’s GTFS and OSM data. GTFS datasets are collected from the New York City Department of Transportation (DOT) and Metropolitan Transportation Authority (MTA), recording schedule and operation details of over 400 transit routes covering all transit modes, i.e. bus, subway, commuting rail, shuttle bus, and ferry, in New York City’s all five boroughs. For the longitudinal analysis of transit accessibility, we collect GTFS datasets in May 2014, 2016, 2019, and 2022 to capture the temporal evolution of New York City’s transit network (Table S3). Then the OTP engine searches for the fastest path by these public transit routes between all 39,557,810 origin–destination block group pairs. We specify the origins and destinations as block groups’ centroids and the departure times as every 10 min between 7:00 AM and 9:00 AM on a weekday. The door-to-door travel time by public transit between each pair of block groups *i* and *j*, denoted as $t_{i,j}$, is averaged on all 13 departure times. This strategy of multiple departure times can produce robust quantification by reducing potential errors to no more than 2.5% (75). The assumption of using centroid-to-centroid transit time may oversimplify the analysis when there are large variations within block group areas. This impact in our study is minimal since block groups in New York City have consistently small sizes with an average radius of 200 m (see Supplementary Notes S1 for details).

For each block group *i*, we then derive its accessible block groups within a time threshold *t*, $N_{i}(t)=\left\{j|t_{i,j}\leq t\right\}$, and the corresponding border $\partial N_{i}(t)=\left\{j||t_{i,j}-t|\leq \text{2.5}\min\right\}$ from queried travel times.

Based on queried travel times between block groups, we measure block groups’ transit accessibility to urban resources. We first measure the accessible radius $r_{i}(t)$ of block group *i*, which is defined as the average spatial distance to all other block groups that can be accessed within [t−2.5, t+2.5] min,


(1) $$r_{i}(t)=\frac{1}{\left|\partial N_{i}(t)\right|}\sum_{\;j\in \partial N_{i}(t)}d_{i,j},$$

where $d_{i,j}$ is the spatial distance between *i* and *j*. The accessible radius r(t) reflects the block group’s potential accessibility to urban opportunities via public transit, defined as the mean distance within which destinations can be reached under a given travel-time threshold. A higher r(t) indicates better accessibility. This metric is conceptually similar to the market access measure used in urban and trade economics (76, 77), though here we adopt the threshold-based cumulative opportunity approach that is more common in the transportation and planning literature.

We then sum up the number of employment opportunities, banks, healthcare facilities, parks, and schools in $N_{i}(t)$ as $O_{i}(t)$, the accessible resources of *i* within *t*:


(2) $$O_{i}(t)=\sum_{\;j\in N_{i}(t)}o_{j},$$

where $o_{j}$ represents the number of urban resources in block group *j*. We collect the number of job opportunities in each block group from the LEHD Origin-Destination Employment Statistics (LODES) 2019 dataset (78), and the location information of four typical facilities, i.e. banks, healthcare, parks, and schools from SafeGraph’s Places dataset (79) (see Table S4 for facility categorization). There are six transit accessibility metrics, namely the accessible radius $r_{i}(t)$, accessible job opportunities, and four metrics of accessible essential facilities (banks, healthcare facilities, parks, and schools). We specify the travel time threshold *t* as 60 min. This threshold is approximate with the average one-way commute time of New York City’s commuters (53 min) (80). The distributions of transit accessibility are illustrated in Fig. S2.

To compare accessibility among populations of different races, the transit accessibility for a specific race is calculated as the average over all block groups, weighted using the population of that race for each transit-oriented block group (81). Two-sided t-tests are applied to test differences in average accessibility metrics between racial groups. Demographics of block groups are collected from American Community Survey (ACS) 5-Year Estimates data. Detailed statistics are listed in Tables S1 and S2.

We estimate linear regression models that control block groups’ socioeconomic variables. The dependent variable of each model is one of the six transit accessibility metrics. Independent variables are race, income percentile in the city, poverty rate, unemployment rate, car ownership rate, the share of commuters using public transit, foreign-born rate, and housing value. Each block group is disaggregated into four data samples with a categorical race variable taking “white,” “Hispanic,” “black,” or “other” and socioeconomic variables identical with the block group. Each sample is weighted with the population of the corresponding race in the block group during the regression. We take “white” as the base reference level of race and test the significance of “Hispanic” and “black”’s regression coefficients by two-sided *t*-tests. To ensure valid inference, all regressions report heteroscedasticity-robust standard errors clustered at the census tract level, which allows for arbitrary correlation across block groups within the same tract and provides a conservative correction for potential spatial dependence (see Supplementary Note S2 for more details on regression settings).

### M2. Experienced segregation in accessible block groups

Extensive research has revealed urban dwellers experience segregation in their daily lives using dynamic mobility records and residential distributions (30, 38, 57). As a key function of facilitating mobility, we further integrate urban transit networks with segregation and propose a metric to characterize segregation based on transit accessibility. Specifically, we define block groups’ empirical and equitable accessible block groups to quantify the effect of transit systems on residential segregation. Given a time threshold *t*, the empirical accessible block groups of a block group consists of all destination block groups accessible within *t*. The equitable accessible block groups under *t* is the empirical accessible block groups combined with all destination block groups within average radius d(t), which is the average spatial distance of all trips in the city with travel time equals *t* (the orange line in Fig. 3B). Figure 3B depicts the trend of d(t) over *t*. The equitable accessible block groups represents what could be accessed via an equitable and isotropic transit network that disregards differences in demographic backgrounds or travel directions. The experienced segregation index *S* of accessible block groups is then defined as the Kullback–Leibner (KL) divergence (82) of its racial composition from the overall racial composition in the city (32.3% non-Hispanic White, 22.2% Hispanic, 29.0% non-Hispanic Black, and 16.5% others, see Fig. S7). The relative difference between *S* of equitable and empirical accessible block groups is regarded as the proportion of segregation that can be mitigated by equitable transit networks.

### M3. Measuring mobility behavior consequences

We compute block groups’ mobility behaviors by SafeGraph’s mobility dataset (40) which records the monthly visit counts between all block groups and all points of interest (POIs) from March to May 2019 derived from a panel of opted-in mobile devices. There are over 700,000 mobile devices and over 66 million visits recorded (Table S5). This dataset covers a relatively comprehensive set of POIs with accurate mobility data management. Individual visits are aggregated to a block group level under stringent privacy regulations. From the aggregated visit information, we compute block groups’ radius of gyration and average travel distance to banks, healthcare facilities, parks, and schools from their visit records. A block group’s radius of gyration is defined as the average distance from it to all POIs visited. The average travel distance to a specific facility category is calculated based on the block group’s visits to POIs belonging to that category within the city limit. All metrics are weighted by the count of visits originating from the block group to the POIs (see Supplementary Note S3 for details). The distributions of real-world behaviors are illustrated in Fig. S9.

To explore how racial disparities in transit accessibility are statistically associated with disparities in real-world behaviors, we use the “mediate” function of the “mediation” R package (83) to conduct mediation analysis (41). This framework estimates the proportion of observed differences between white and black populations in mobility outcomes that can be statistically accounted for by racial differences in transit accessibility. We take the racial background as the independent variable *Z*, real-world behavior as the dependent variable *Y*, and transit accessibility as the mediation variable *M*. We first estimate a linear regression model of *Y* on *Z* and document the regression coefficient of “black” over “white” as $c^{\prime }$. The regression coefficient of *M* over *Z* is recorded as *a*. Finally, a regression model of *Y* over both *Z* and *M* is fitted, where coefficients of *Z* and *M* are *c* and *b*, respectively. All regression models include covariate variables Fig. 2I along with the rates of children residing in nonmarried-couple families, given the potential impact of family structure on mobility behaviors (84) (Fig. S11). The mediation proportion is the ratio of the mediation effect to the sum of the mediation effect and direct effect ab/(c+ab), representing the share of the total association that is statistically linked to transit accessibility (see Supplementary Note S3 for more details).

### M4. Examining transit accessibility in public housing districts

Racial inequality in transit accessibility requires effective mitigation policies targeting specific disadvantaged areas. We select public housing developments as a representative case of districts with low levels of government investments and high minority concentration. The location information of public housing developments is provided by New York City Open Data (85). Block groups containing at least one public housing development are identified as public housing block groups $N_{\mathrm{ph}}$. Others are nonpublic housing block groups $N_{\mathrm{nph}}$.

We calculate the differences in average transit accessibility of public housing and nonpublic housing block groups $\Delta_{Y}=\frac{1}{\left|N_{\mathrm{nph}}\right|}\sum_{\;j\in N_{\mathrm{nph}}}Y_{j}-\frac{1}{\left|N_{\mathrm{ph}}\right|}\sum_{i\in N_{\mathrm{ph}}}Y_{i}$, where *Y* denotes a transit accessibility metric. To examine the feasibility of mitigating accessibility inequality through increased investment in urban infrastructure and enhanced management in underprivileged areas, we propose four simulation scenarios as outlined below. First, the public housing-improved (PH-improved) scenario, where the accessibility of public housing block groups is raised to the same level as nonpublic housing block groups by adding $\Delta_{Y}$ to each public housing block group’s accessibility $Y_{i}$ that is, ${\hat{Y}}_{i}=Y_{i}+\Delta_{Y}$. Second, the bus speed-up (BSU) scenario, where bus routes in Bronx, Brooklyn, and Queens speed up by 10% (50). Third, the Interborough Express (IBX) scenario, which entails the realization of the Interborough Express project (51) proposed by MTA. that connects underserved areas of Brooklyn and Queens. Fourth, the Triborough Express (TBX) scenario proposes the design of two new subway lines connecting Bronx, Brooklyn, and Queens, formulated using a mathematical programming algorithm for network expansion (52) (see Supplementary Note S4 for details). We calculate the accessibility gaps between ethnic minorities and the white population under both empirical and simulated scenarios to evaluate the potential reduction in accessibility gaps through these proposed strategies.

## Supplementary Material

pgag025_Supplementary_Data

## Data Availability

The American Community Survey data are collected from the US Census Bureau (https://www.census.gov/data/developers/data-sets/acs-5year.2019.html). The geographic boundaries of census block groups are obtained from the US Census Bureau 2010 Census Block Maps (https://www.census.gov/geographies/reference-maps/2010/geo/2010-census-block-maps.html). The General Transit Feed Specification of New York City is obtained from the Open Mobility Data platform (https://transitfeeds.com/). The road network and associated variables were obtained from OpenStreetMap data (https://www.openstreetmap.org). Distribution of job opportunities are collected from the LEHD (Longitudinal Employer-Household Dynamics) Origin-Destination Employment Statistics dataset (https://lehd.ces.census.gov/data/). The SafeGraph mobility data and POI data can be requested from the company through the SafeGraph Data for Academics program (https://www.safegraph.com/academics/). The availability of these data is under strict restriction under the Data License Agreement of SafeGraph. The data are used under the license for this study and are not publicly available. Locations of public housing developments are provided by the New York City Housing Authority (https://data.cityofnewyork.us/Housing-Development/NYCHA-Public-Housing-Developments-Map/npwq-dpkb). GTFS data for the Interborough Express are collected from Zenodo (86). Historic Home Owners’ Loan Corporation (HOLC) maps are obtained from the Mapping Inequality Project at the University of Richmond (https://dsl.richmond.edu/panorama/redlining). The code used in this research can be found on GitHub (https://github.com/tsinghua-fib-lab/TransitIneq).

## Preprints

A preprint of this article is published at https://doi.org/10.21203/rs.3.rs-3485896/v1.
