# Supplementary material for: Quantifying racial inequality in transit access across New York City
Source: PNAS Nexus. 2026 May 19;5(5):pgag025. doi: 10.1093/pnasnexus/pgag025 (PMC13185150; doi:10.1093/pnasnexus/pgag025)
Supplement: pgag025_Supplementary_Data [file pgag025_supplementary_data.pdf]

Supplementary Information for Quantifying racial inequality in transit access across New York City

Contents

S1 Detailed descriptions of data 1
S2 Quantifying transit accessibility 2
S3 Measuring mobility behavior consequences 4
S4 Simulation scenarios 5

Supplementary Notes

S1 Detailed descriptions of data

Research area and unit

Our study focuses on census block groups (CBGs), which we refer to as "block group" in the Main Text, in New York City. The geographic boundaries of census block groups are obtained from U.S. Census Bureau 2010 Census Block Maps [1]. The boundary of New York City is retrieved from NYC Open Data [2]. There are 6,290 block groups in consideration.

CBGs are geographic units containing about 600 to 3,000 people defined by the U.S. Census Bureau. When quantifying transit accessibility, we compute travel times between centroids of CBGs. Large disparities in CBG sizes could introduce errors in the quantification when actual travels originate distantly away from the centroid. Although CBGs in rural areas can be very large, the sizes of CBGs are consistently small in urban areas. In New York City, the average and standard deviation of block group radius are 200 and 59 meters, respectively. This relatively small and consistent size has minimal impact on the analysis. In addition, many studies on urban sciences and transportation geography are also conducted on the CBG level [1, 11, 22], showing no inconsistency issues. Therefore, it is rational to select the census block group as the unit of our analysis.

Block group characteristics

For block group demographics, we collect the American Community Survey (ACS) 5-Year Estimates data from the U.S. Census Bureau [3]. To study racial inequality in transit accessibility, we mainly focus on the following block group demographics - total population, number of households, non-Hispanic white population, non-Hispanic black population, Hispanic population, median household income, population in poverty, unemployment population, the population of commuters taking public transit, number of households having at least one vehicle, population of foreign-born persons, median housing value.

We calculate the average public transit usage rate and household car ownership rate in the top 500 populated U.S. cities. City boundaries are collected from Census Bureau's TIGER geographic database [4]. We group cities into bins of population sizes with ranges of 0.6, steps of 0.2 in base-10 logarithms. The average public transit usage rate and car ownership rate at each bin show that in the U.S., highly-populated cities tend to have higher usage of public transit infrastructure. Among them, New York City, the most populated city, has the highest usage of public transit, leading the way in the transition to sustainable transport.

Public housing block groups

To investigate the impact of imbalanced investment in infrastructure on observed racial inequality and develop mitigation policies, we take public housing developments as a representative case of areas that have suffered from inadequate investment in recent years. The locations of public housing developments are provided by New York City Housing Authority (NYCHA) [5].

[1] https://www.census.gov/geographies/reference-maps/2010/geo/2010-census-block-maps.html
[2] https://data.cityofnewyork.us/City-Government/Borough-Boundaries/tqmj-j8zm
[3] https://www.census.gov/programs-surveys/acs
[4] https://www.census.gov/geographies/mapping-files/time-series/geo/carto-boundary-file.html
[5] https://data.cityofnewyork.us/Housing-Development/Map-of-NYCHA-Developments/i9rv-hdr5

There are 277 public housing developments located in 507 different block groups (some of the developments consist of buildings in multiple block groups). These block groups are identified as public housing block groups. The other 5,783 block groups are non-public housing block groups. We compare the demographics of public housing block groups and non-public housing block groups in Table S2.

S2 Quantifying transit accessibility

In this section, we describe in greater detail the process through which we quantify the transit accessibility of each block group and corresponding racial inequality. We first build a transit network in OpenTripPlanner [15] with transit routes operation information and road networks of New York City. Then we acquire pairwise door-to-door travel time between block groups with OpenTripPlanner's API. Finally, we calculate transit accessibility based on travel times and the distribution of essential facilities and job opportunities in NYC block groups.

Building transit network

We use OpenTripPlanner (OTP), an open-source multi-modal trip planner built upon Java that focuses on travel by scheduled public transit. It builds its representation of the transit network from open data in open standard file formats (here we use GTFS and OpenStreetMap). Then it provides a routing algorithm to automatically retrieve the minimum travel time between each two points on the built graph by any desired travel mode.

We mainly collect two data sources to construct the transit network. An OpenStreetMap (OSM) road network covering New York City and the General Transit Feed Specification (GTFS) datasets provided by transit agencies in New York City, including the Metropolitan Transportation Authority (MTA) and the Department of Transportation (DOT). The OSM road network is extracted from BBBike.org [6]. It provides information on walking distances to transit stops/stations.

The GTFS data are collected from Open Mobility Data [7]. Transit agencies provide detailed schedules (transit feeds) of their transit routes, including the departure and arrival time at each stop and route transfer information. In our analysis, we use the transit feeds in May 2014, 2016, 2019, and 2022. Take the 2022 snapshot as an example, there are 11 agencies providing 429 transit routes in total. We list the names of agencies and corresponding transit routes in Table S3. We use the "build" command of OpenTripPlanner to build a graph consisting of OSM road network and transit feeds, which produce a .obj file that can be further analyzed.

Calculating travel times

After running an OTP graph object on the local server, we call the OTP Planner Resource API [8] by Python package "requests". Specifically, in each request, we provide the geographic coordinates of the origin and destination points, the date, the starting time, and the travel mode. Then the OTP engine will calculate the travel details of the route that takes minimum time from the origin to the destination, including the time taking transit as well as walking time. We specify the date as a weekday (May 2nd in 2014, May 3rd in 2016, 2019, and 2022) and departure times as every 10 minutes from 7 AM to 9 AM (7:00, 7:10, ..., 8:50, 9:00). This interval can reduce estimation errors to no more than 2.5% [23]. The travel mode is set as "WALK, TRANSIT" in the API to query travels by urban transit.

For all block groups in New York City, we query its travel time to each of the other 6,289 block groups. The origin and destination are set as the centroids of both block groups. There are 39,557,810 origin-destination block group pairs in total. We denote the door-to-door travel time by transit from block groups i to block group j as $t_{i,j}$, which is averaged on all 13 departure times.

Based on the queried travel times, we can derive the accessible block groups of a block group within given time thresholds. We define the accessible block groups of block group i within time threshold t as

$$\mathscr{N}_i(t) = \{j | t_{i,j} <= t\}.$$

Then, the corresponding border of an accessible block groups $\mathscr{N}_i(t)$ is

$$\partial \mathscr{N}_i(t) = \{j | t_{i,j} = t\},$$

or more broadly defined as

$$\partial \mathscr{N}_i(t) = \{j | t - 2.5 <= t_{i,j} <= t + 2.5\}.$$

[6] https://extract.bbbike.org/
[7] https://transitfeeds.com/l/91-new-york-ny-usa
[8] http://dev.opentripplanner.org/apidoc/1.0.0/resource_PlannerResource.html

Based on the above definitions, we measure block groups' transit accessibility to urban block groups (accessible radius), job opportunities, and essential facilities. Figure S2 depicts the distribution of these metrics. Please refer to *Methods - Quantifying transit accessibility* in the Main Text for details.

Alternative accessibility measurement

In addition to the above cumulative accessibility metrics, another widely-used metric of accessibility in the transportation geography literature is gravity-based accessibility measurement [7, 10], which incorporates a time-decay factor $f(\cdot)$ into accessibility evaluation by the following equation,

$$A_i = \sum_j o_j f(C_{ij}),$$

where C_{ij} is the transit time from block group i to j and $f(\cdot)$ is the impedance function. Here we take $f(\cdot)$ as a log-logistic decay-cumulative density function [17] and fit the parameters with transit times queried in 2019. We obtain

$$\hat{f}(C_{ij}) = \frac{1}{1 + (\frac{C_{ij}}{69.812\text{min}})^{4.478}}.$$

Then we can compute the gravity-based accessible resources (job opportunities and essential facilities) with corresponding resource distribution o_j and the fitted impedance function $\hat{f}(\cdot)$. Racial inequality in gravity-based accessibility metrics is depicted in Figure S3.

Previous studies have shown that cumulative accessibility measures under the average commute time, the transit accessibility metrics in the Main Text, can be used as an effective substitute for gravity-based measures [7, 10, 20]. Moreover, gravity-based measures are more theoretically complicated and potentially biased due to the various forms of the decaying function [8]. In comparison, cumulative measures are more intuitive and concise. Therefore, we report results in the Main Text based on cumulative accessibility measures.

Quantify accessibility inequality

To quantify inequality in transit accessibility among populations of different races, we first calculate the weighted average accessibility of each race among all transit-dependent block groups with the following equation described by Rowangould *et. al.* [19],

$$Y(\text{race}) = \frac{\sum_{i=1}^{N} p_i(\text{race}) Y_i}{\sum_{i=1}^{N} p_i(\text{race})},$$

where Y_i is the accessibility for block group i and $p_i(\text{race})$ is the population of that race in i.

To further rule out the confounding effects of socioeconomic variables, we estimated weighted linear regression models with transit accessibility metrics as the dependent variable Y.

$$Y_i = \beta_0 + \beta_{\text{Hispanic}} Z_{\text{Hispanic}} + \beta_{\text{black}} Z_{\text{black}} + \beta_{\text{other}} Z_{\text{other}} + \beta_X^T \mathbf{X}_i + \varepsilon_i$$

Here we segment each block group into four samples in the regression, each representing the population of one specific race (white, Hispanic, black, and others) in that block group. We use a dummy variable Z in models to represent the racial group of each sample and take "white" as the reference level. The coefficients of the remaining groups ($\beta_{\text{Hispanic}}, \beta_{\text{black}}, \beta_{\text{other}}$) represent their expected difference compared with the white population. We include the following covariates $\mathbf{X}_i$ in the models that may contribute to transit accessibility, income level (represented as the percentile of a block group in the city), poverty rate, unemployment rate, car ownership rate, public transit usage rate, foreign-born rate, and median housing value (in million dollars).

The models are estimated by the WLS function in the Python package "statsmodels" [21], where each sample is weighted by its corresponding population. To ensure conservative inference given repeated block group-level observations and potential spatial dependence, we compute heteroskedasticity-robust standard errors clustered at the census tract level. This specification allows arbitrary correlation among block groups within the same tract, providing robustness to both heteroskedasticity and tract-level spatial autocorrelation. We report the regression coefficients β's, their corresponding standard errors and significance levels, model R squared value, and F-statistic in the manuscript. All models have significant F-statistics.

We employ regression analysis spanning four years (2014, 2016, 2019, and 2022) to examine the temporal dynamics of accessibility gaps between ethnic minorities and the white population (Figure S5). Figure S6 depicts shifts in gaps of six transit accessibility metrics from 2014 to 2022 with socioeconomic characteristics controlled. Additionally, we deconstruct the trajectory of these disparities into the influences of residential sorting and transit network evolution. First, we construct a supply-only counterfactual in which block group demographics are fixed at their 2014 ACS values while transit networks evolve.

This scenario removes demographic change and isolates the contribution of network expansion or underinvestment. The dashed lines in Figure S6 represent the accessibility gaps under this counterfactual. Second, we construct a sorting-only counterfactual in which the 2014 transit network is held fixed while racial demographics evolve according to subsequent ACS estimates. This scenario removes changes in transit provision and isolates the contribution of residential sorting and demographic redistribution. The dotted lines in Figure S6 represent accessibility gaps under this counterfactual. By comparing the observed gaps with these two counterfactual scenarios, we can partially attribute the widening disparities to network-side factors, demographic sorting, and their interaction. Notably, accessibility gaps are found to increase under both counterfactuals, indicating that both supply and sorting processes independently contribute to the persistence and escalation of racialized inequality in transit access.

Finally, we investigate the historical roots of these accessibility disparities by overlaying current transit accessibility with neighborhood grades from the Home Owners' Loan Corporation (HOLC) security maps of the late 1930s. These grades summarize contemporaneous lending-risk perceptions produced by local real-estate professionals and are widely used as an archival record of discriminatory appraisal logics. HOLC classified neighborhoods from "A" (Best) and "B" (Still Desirable) to "C" (Declining) and "D" (Hazardous), capturing contemporaneous neighborhood risk perceptions shaped by race and poverty and widely used as an archival record of discriminatory appraisal logics. We group block groups historically graded A/B as advantaged areas and those graded C/D as disadvantaged areas. We compare the population-weighted average transit accessibility across these two groups in Figure S14.

S3 Measuring mobility behavior consequences

Mobility dataset

World leaders recognized universally at the 2012 United Nations Conference on Sustainable Development that transit and mobility are central to sustainable development [13]. To link transit accessibility with human mobility in cities, we adopt the SafeGraph's Patterns dataset [9]. This large-scale dataset uses a panel of opted-in mobile applications to record the number of visit counts to each point of interest (POI), or place in the United States, and the corresponding origin block group of visitors. We use the dataset from March to May 2019, counting total visit counts between each block group-POI pair. We list the respective number of devices and visit counts in each month in Table S5. There are over 700,000 devices and over 66 million visits recorded.

This mobility dataset provided by SafeGraph is widely utilized in previous studies to understand patterns of human mobility [16, 5, 18], which consolidates the feasibility of applying it to quantify the impact of transit accessibility on mobility behaviors. Specifically, previous studies have demonstrated the high coverage and representativeness of this dataset [12, 1]. It has a relatively balanced sample rate across demographics and a reasonable coverage rate with a small sampling bias. Based on the above observations, we adopt the SafeGraph mobility dataset as a good approximation of large-scale real-world mobility behaviors.

Mobility behaviors

For each block group, we first calculate its radius of gyration over the course of three months using the following formula,

$$r_g(i) = \sqrt{\frac{\sum_j v_{i,j} d_{i,j}^2}{\sum_j v_{i,j}}},$$

where $v_{i,j}$ denotes the number of visits originated from block group i to POI j, and $d_{i,j}$ denotes the distance from the centroid of block group i to POI j. Following previous works [25], We exclude the top 1% of distances to eliminate possible biases from extremely long-distance travel. The radius of gyration illustrates the range of the block group's daily activities. Although both the radius of gyration r_g and accessible facilities $O(t)$ are related to POIs, they originate from distinct analytical perspectives. The radius of gyration r_g is a behavioral metric, centering on the realized visits to all POIs. By contrast, accessible facilities $O(t)$ serve as metrics of accessibility that quantify the number of specific facilities that can be accessed by a block group through the transit network. Therefore, the distinct focuses of these concepts distinguish them.

We also calculate block group i's average travel distance to facility categories C:

$$d_C(i) = \sqrt{\frac{\sum_{j \in C} v_{i,j} d_{i,j}^2}{\sum_{j \in C} v_{i,j}}},$$

which is the radius of gyration to all POIs belonging to category C. Here, C is one of the four categories we are interested in, *i.e.*, banks, healthcare facilities, parks, and schools. Figure S9 shows the distribution of the block group's radius of gyration r_g and average travel distance d_C. Figure S10 shows the discrepancies in mobility behaviors among block groups with different racial backgrounds.

[9] https://docs.safegraph.com/docs/monthly-patterns

Details of mediation analysis

We adopt mediation analysis [14] to estimate the percentage of racial disparities in mobility behaviors that can be statistically accounted for by racial gaps in accessibility. Since the SafeGraph data is collected in 2019, we use 2019 accessibility metrics in this analysis. Specifically, we estimate three regression models over three sets of variables, *i.e.*, the independent variable Z - race background, the dependent variable Y - mobility pattern, and the mediation variable M - accessibility metric. We first regress Y over Z:

$$Y = \beta_{01} + c'Z + \beta_{X1}^T \mathbf{X} + \varepsilon_1,$$

where $\mathbf{X}$ is the set of covariate variables as introduced in Section S2. Since family structure, which varies across different racial groups (Supplementary Figure S1c), may impact activity choices [6] (Supplementary Figure S11), we further include the rates of children residing in non-married-couple families into $\mathbf{X}$. Then we regress mediation variable M over Z:

$$M = \beta_{02} + aZ + \beta_{X2}^T \mathbf{X} + \varepsilon_2.$$

Finally we regress Y over both Z and M:

$$Y = \beta_{03} + cZ + bM + \beta_{X3}^T \mathbf{X} + \varepsilon_3.$$

The "total effect" from Z to Y is $c' = c + ab$, the direct effect is c, and the indirect effect (mediation effect) is ab. Then, $\frac{ab}{c+ab}$ is defined as the mediation proportion. We take "white" as the control level and "black" as the treatment level for the independent variable Z. We report the dependent variable, mediation variable, regression coefficients, and the mediation proportion in Table S6 for each real-world behavior that is significantly mediated by transit accessibility.

S4 Simulation scenarios

We simulate four scenarios to evaluate the potential mitigation effect of transit infrastructure policies on current racial gaps in transit accessibility. The empirical gap of accessibility metric Y is calculated as $(Y(\text{white}) - Y(\text{Hispanic}))/Y(\text{Hispanic})$ and $(Y(\text{white}) - Y(\text{black}))/Y(\text{black})$. In each of the following scenarios, we calculate corresponding simulated accessibility $\hat{Y}$ to derive the simulated accessibility gaps.

Public housing-improved scenario

In this scenario, we assume that public housing block groups have the same level of transit accessibility as other block groups, which is implemented by adding Δ_Y to each public housing block group's accessibility Y, *i.e.*, $\hat{Y}_i = Y_i + \Delta_Y$. Here $\Delta_Y = \frac{1}{|N_{nph}|}\sum_{j \in N_{nph}} Y_j - \frac{1}{|N_{ph}|}\sum_{i \in N_{ph}} Y_i$, representing the disparity between the average accessibility of public housing and non-public housing block groups. The accessibility metrics for non-public housing block groups remain unaltered.

Bus speed-up

The New York City Department of Transportation initiated the Better Bus Action Plan in 2019 [24], which focused on improving bus speeds citywide by 25% and reversing downward bus ridership trends. Moreover, according to the Bus Network Redesign project of NYC MTA [4], Bronx, Brooklyn, Queens, and Staten Island are planning to speed up their bus routes, which can be achieved by prioritizing buses on targeted corridors, increasing frequencies, improving connections, and balancing stop spacing. Based on current planning prospects, we propose a bus speed-up scenario where bus speeds in three boroughs (Bronx, Brooklyn, and Queens) are increased by 10%. Specifically, we use the "set_trip_speed" function of R package "gtfstools" [9] to alter the speeds of bus routes.

Interborough express

MTA is planning an Interborough Express project [3], which is a rapid light rail system that will connect currently underserved areas of Brooklyn and Queens. This line will connect with up to 17 subway lines and the Long Island Rail Road, providing a fast and convenient transit option in underserved locations where more than a third of residents are below the federal poverty line. We collect the GTFS data of the Interborough Express from Zenodo [27], where the departure interval in the morning peak is 5 minutes. The route and station location of Interborough Express are depicted in Figure S13.

Triborough express

An essential issue of urban transportation is the expansion of rapid transit systems based on existing lines. In New York City, most subway lines are concentrated in Manhattan (Figure S13), with few connections between the Bronx, Brooklyn, and Queens. In history, there have been plans such as the Triboro RX [2], which aimed to connect three boroughs but were discontinued due to specific reasons.

Following the idea of triborough connections, we employ a mathematical programming algorithm to develop new subway lines in New York City [26]. The algorithm aims to optimize the number of travel demands covered by the subway network. We compute the origin-destination (OD) matrix between census tracts in New York City from the SafeGraph dataset and take the OD flow as travel demands. Travel demands are considered be fulfilled if both the origin and destination tract have subway stations located. We select census tracts with accessible radius lower than 10.5 kilometers as locations of candidate stations. We generate two lines by the algorithm (red lines in Figure S13) which have extensive connections with existing lines. We refer to these generated lines as "Triborough Express" and prepare corresponding GTFS data. The departure interval in the morning peak is set as 4 minutes.

References

[1] Timur Abbiasov, Cate Heine, Sadegh Sabouri, Arianna Salazar-Miranda, Paolo Santi, Edward Glaeser, and Carlo Ratti. "The 15-minute city quantified using human mobility data". In: *Nature Human Behaviour* (2024), pp. 1–11.

[2] Regional Plan Association. *Third Regional Plan Summary*. 1996.

[3] Metropolitan Transportation Authority. *Interborough Express*. 2024. URL: https://new.mta.info/project/interborough-express.

[4] The Metropolitan Transportation Authority. *Bus Network Redesign*. 2023. URL: https://new.mta.info/project/bus-network-redesign.

[5] Serina Chang, Emma Pierson, Pang Wei Koh, Jaline Gerardin, Beth Redbird, David Grusky, and Jure Leskovec. "Mobility network models of COVID-19 explain inequities and inform reopening". In: *Nature* 589.7840 (2021), pp. 82–87.

[6] Yingling Fan. "Household structure and gender differences in travel time: spouse/partner presence, parenthood, and breadwinner status". In: *Transportation* 44 (2017), pp. 271–291.

[7] Karst T Geurs and Bert Van Wee. "Accessibility evaluation of land-use and transport strategies: review and research directions". In: *Journal of Transport geography* 12.2 (2004), pp. 127–140.

[8] Mariana Giannotti, Diego B Tomasiello, and Taina A Bittencourt. "The bias in estimating accessibility inequalities using gravity-based metrics". In: *Journal of transport geography* 101 (2022), p. 103337.

[9] Daniel Herszenhut, Rafael H. M. Pereira, Pedro R. Andrade, and Joao Bazzo. *gtfstools: General Transit Feed Specification (GTFS) Editing and Analysing Tools*. https://github.com/ipeaGIT/gtfstools. 2023.

[10] Bogdan Kapatsila, Manuel Santana Palacios, Emily Grisé, and Ahmed El-Geneidy. "Resolving the accessibility dilemma: Comparing cumulative and gravity-based measures of accessibility in eight Canadian cities". In: *Journal of Transport Geography* 107 (2023), p. 103530.

[11] Alex Karner. "Assessing public transit service equity using route-level accessibility measures and public data". In: *Journal of Transport Geography* 67 (2018), pp. 24–32.

[12] Zhenlong Li, Huan Ning, Fengrui Jing, and M Naser Lessani. "Understanding the bias of mobile location data across spatial scales and over time: a comprehensive analysis of SafeGraph data in the United States". In: *Plos one* 19.1 (2024), e0294430.

[13] Björn-Ola Linnér and Henrik Selin. "The United Nations Conference on Sustainable Development: forty years in the making". In: *Environment and Planning C: Government and Policy* 31.6 (2013), pp. 971–987.

[14] David P MacKinnon. *Introduction to statistical mediation analysis*. Routledge, 2012.

[15] Malcolm Morgan, Marcus Young, Robin Lovelace, and Layik Hama. "OpenTripPlanner for R". In: *Journal of Open Source Software* 4.44 (2019), p. 1926.

[16] Hamed Nilforoshan, Wenli Looi, Emma Pierson, Blanca Villanueva, Nic Fishman, Yiling Chen, John Sholar, Beth Redbird, David Grusky, and Jure Leskovec. "Human mobility networks reveal increased segregation in large cities". In: *Nature* 624.7992 (2023), pp. 586–592.

[17] Antonio Páez, Darren M Scott, and Catherine Morency. "Measuring accessibility: positive and normative implementations of various accessibility indicators". In: *Journal of Transport Geography* 25 (2012), pp. 141–153.

[18] Sen Pei, Teresa K Yamana, Sasikiran Kandula, Marta Galanti, and Jeffrey Shaman. "Burden and characteristics of COVID-19 in the United States during 2020". In: *Nature* 598.7880 (2021), pp. 338–341.

[19] Dana Rowangould, Alex Karner, and Jonathan London. "Identifying environmental justice communities for transportation analysis". In: *Transportation Research Part A: Policy and Practice* 88 (2016), pp. 151–162.

[20] Manuel Santana Palacios and Ahmed El-Geneidy. “Cumulative versus gravity-based accessibility measures: which one to use?” In: (2022).

[21] Skipper Seabold and Josef Perktold. “statsmodels: Econometric and statistical modeling with python”. In: *9th Python in Science Conference*. 2010.

[22] Mario L Small, Armin Akhavan, Mo Torres, and Qi Wang. “Banks, alternative institutions and the spatial–temporal ecology of racial inequality in US cities”. In: *Nature Human Behaviour* 5.12 (2021), pp. 1622–1628.

[23] Marcin Stępniak, John P Pritchard, Karst T Geurs, and Sławomir Goliszek. “The impact of temporal resolution on public transport accessibility measurement: Review and case study in Poland”. In: *Journal of transport geography* 75 (2019), pp. 8–24.

[24] The New York City Department of Transportation. *Better Buses Action Plan*. 2019. URL: https://www.nyc.gov/html/brt/html/betterbuses/betterbuses.shtml.

[25] Qi Wang, Nolan Edward Phillips, Mario L Small, and Robert J Sampson. “Urban mobility and neighborhood isolation in America’s 50 largest cities”. In: *Proceedings of the National Academy of Sciences* 115.30 (2018), pp. 7735–7740.

[26] Yi Wei, Jian Gang Jin, Jingfeng Yang, and Linjun Lu. “Strategic network expansion of urban rapid transit systems: A bi-objective programming model”. In: *Computer-Aided Civil and Infrastructure Engineering* 34.5 (2019), pp. 431–443.

[27] Hai Yang. *Interborough Express GTFS [Data set]*. 2023. URL: https://doi.org/10.5281/zenodo.8231009.

Supplementary Figures

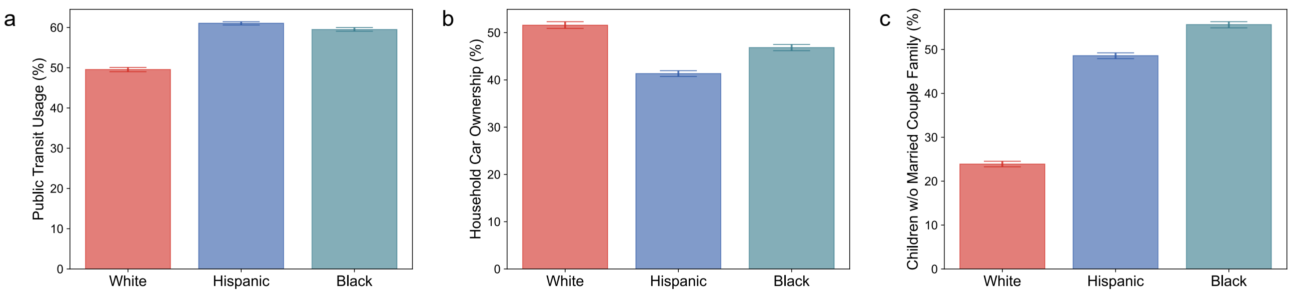

Figure S1. Discrepancies in public transit dependency (a), car ownership (b), and rates of children not living in married-couple families (c) across different racial groups among block groups.

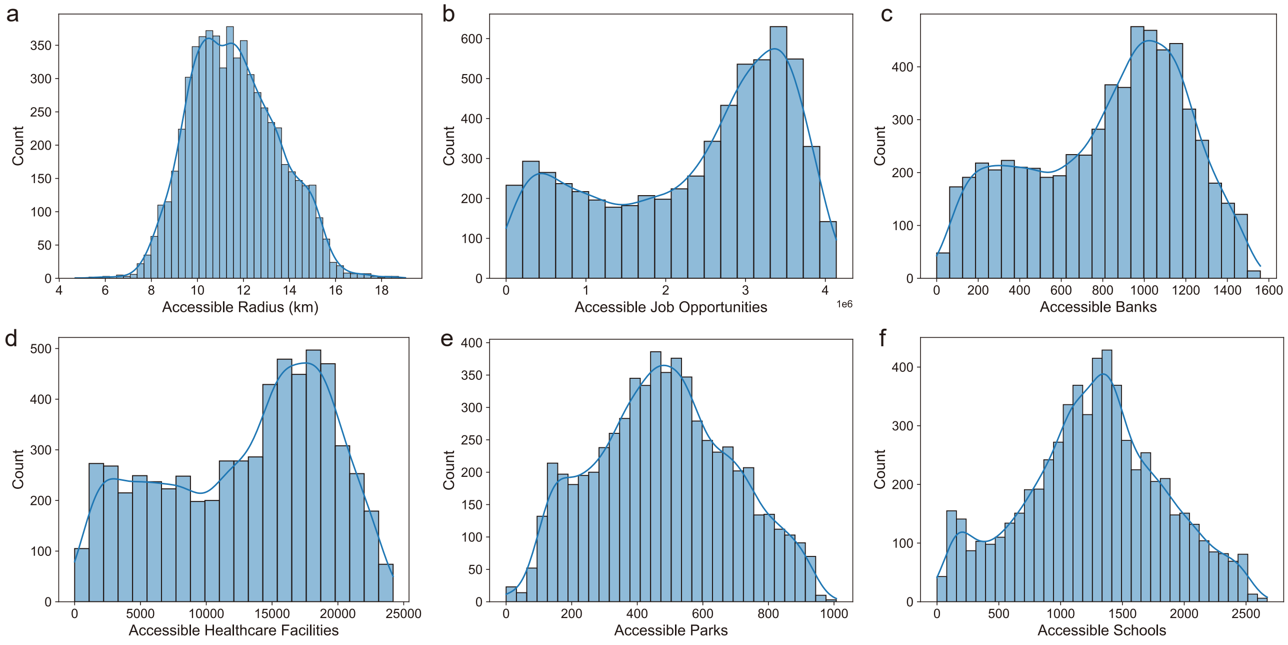

Figure S2. Distributions of block group transit accessibility. (a) accessible radius. (b) accessible job opportunities. (c) accessible banks. (d) accessible healthcare facilities. (e) accessible parks. (f) accessible schools.

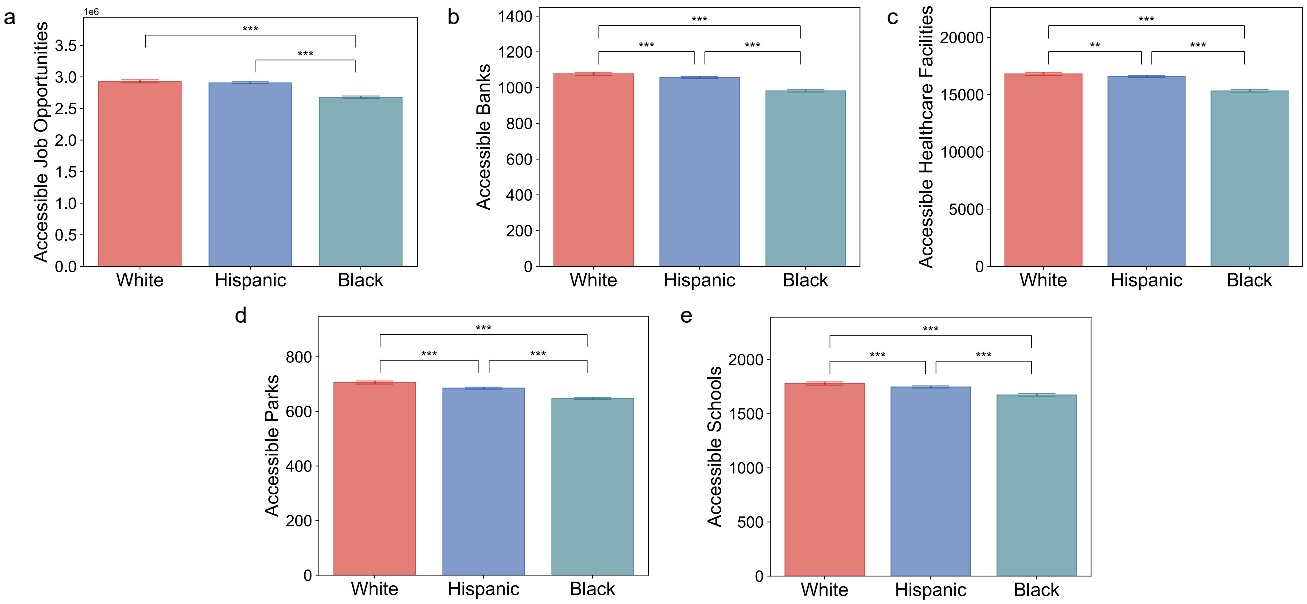

Figure S3. Racial inequality in **gravity-based** accessibility metrics among block groups. The average job opportunities(a), banks(b), healthcare facilities(c), parks(d), and schools(e) of white, Hispanic, and black populations in 2019. Whiskers represent the corresponding 95% confidence intervals. Significance level: *: p<0.05, **: p<0.01, ***: p<0.001.

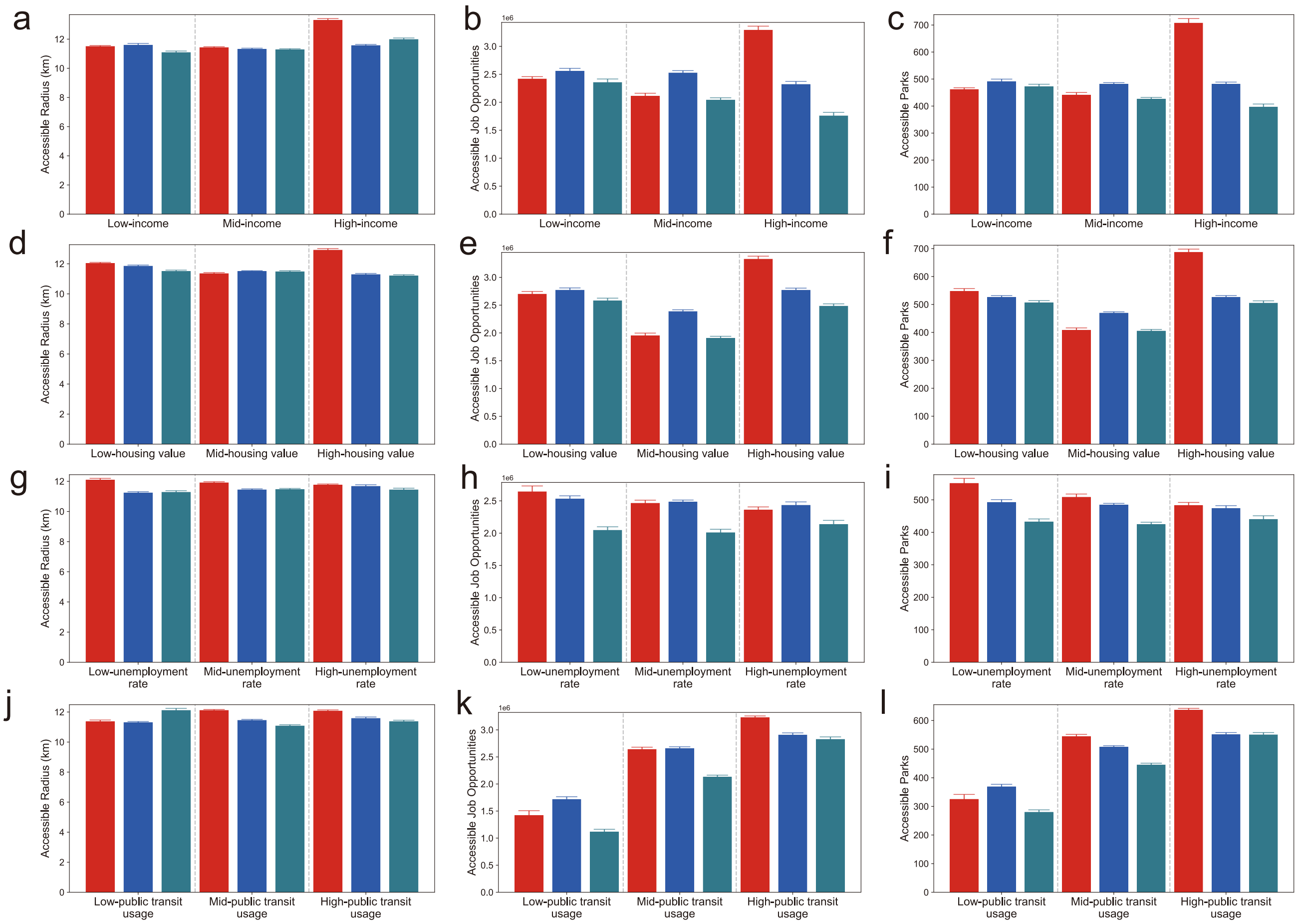

Figure S4. Racial inequality in transit accessibility among block groups with different levels of income(a-c), housing value(d-f), unemployment rate(g-i), and public transit usage(j-l). Low-, mid-, and high- indicate the lowest quartile, second and third quartile, and the highest quartile, respectively. Whiskers represent the corresponding 95% confidence intervals.

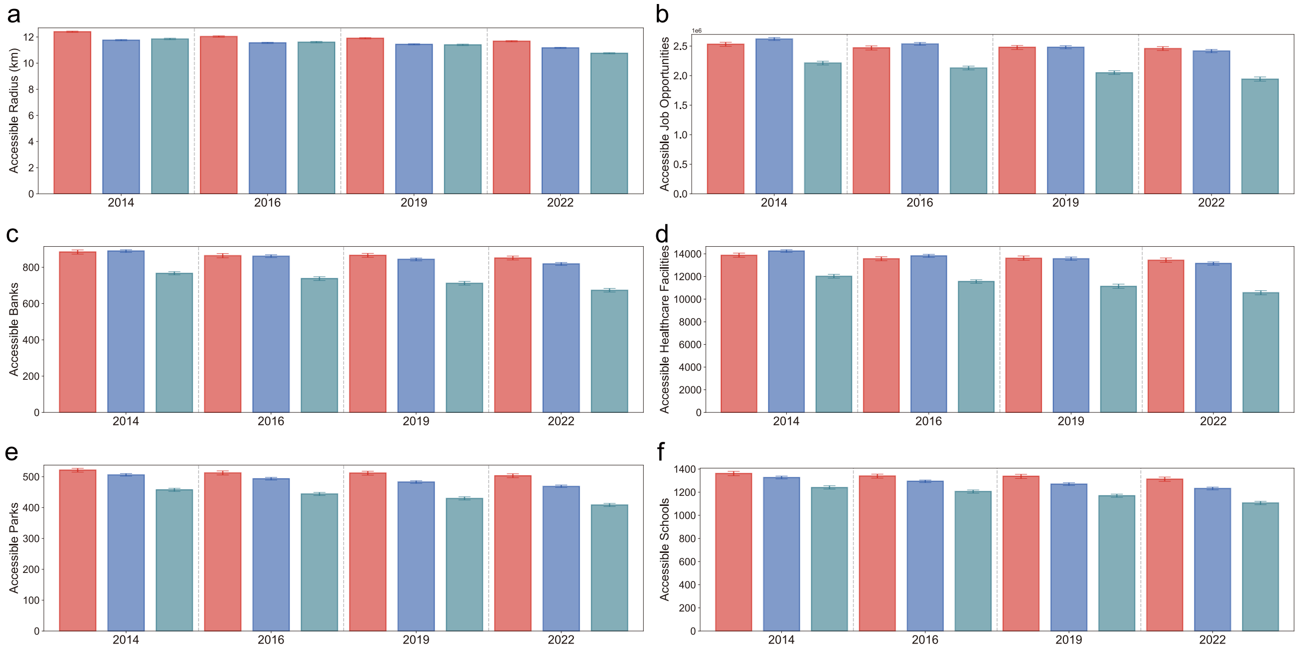

Figure S5. Racial inequality in transit accessibility metrics (accessible radius(a), job opportunities(b), banks(c), healthcare facilities(d), parks(e), and schools(f).) among block groups in 2014, 2016, 2019, and 2022. Whiskers represent the corresponding 95% confidence intervals.

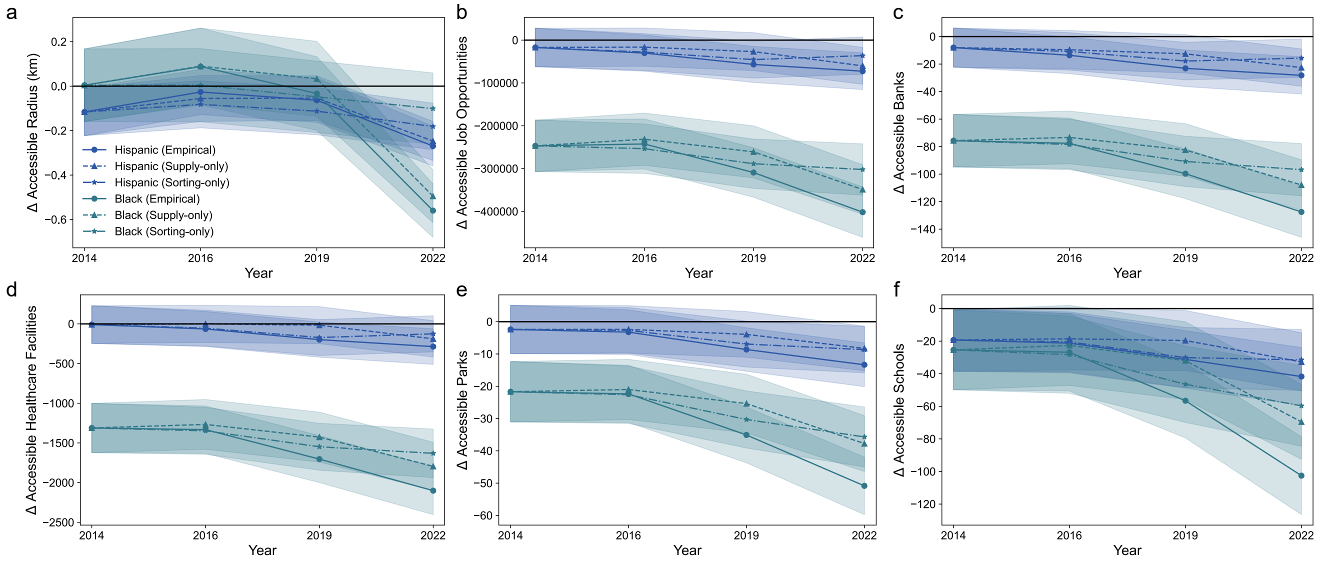

Figure S6. Regression coefficients of Hispanic and black populations in four years over accessible radius(a), job opportunities(b), banks(c), healthcare facilities(d), parks(e), and schools(f). Dashed lines are calculated under two counterfactual scenarios. Supply-only scenario depicts that the demographic distributions in New York City are fixed within this decade. Sorting-only scenario depicts that the accessibility metrics in New York City are fixed within this decade. Shadows represent 95% confidence intervals.

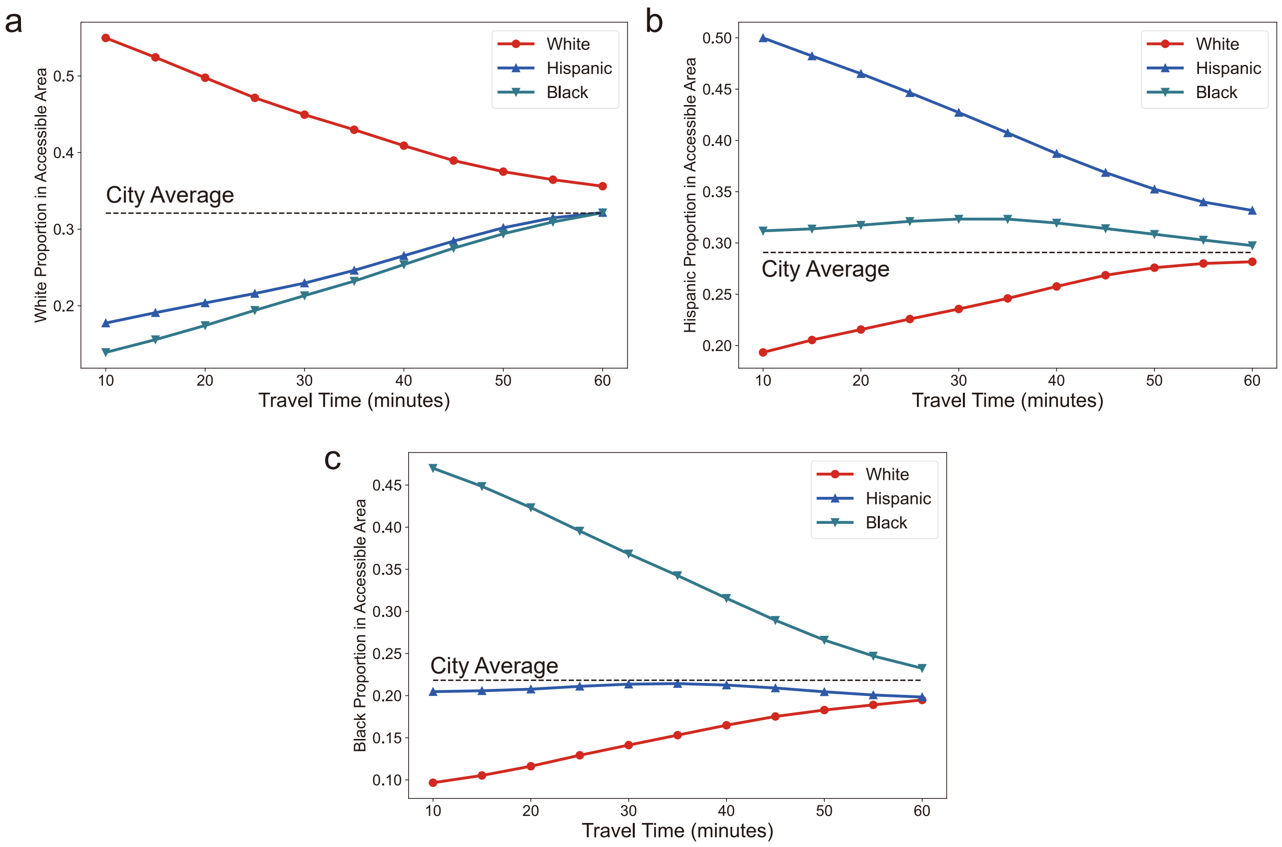

Figure S7. The average proportion of white (a), Hispanic (b), and black (c) residents in real accessible areas within 10 to 60 minutes among block groups of different racial groups.

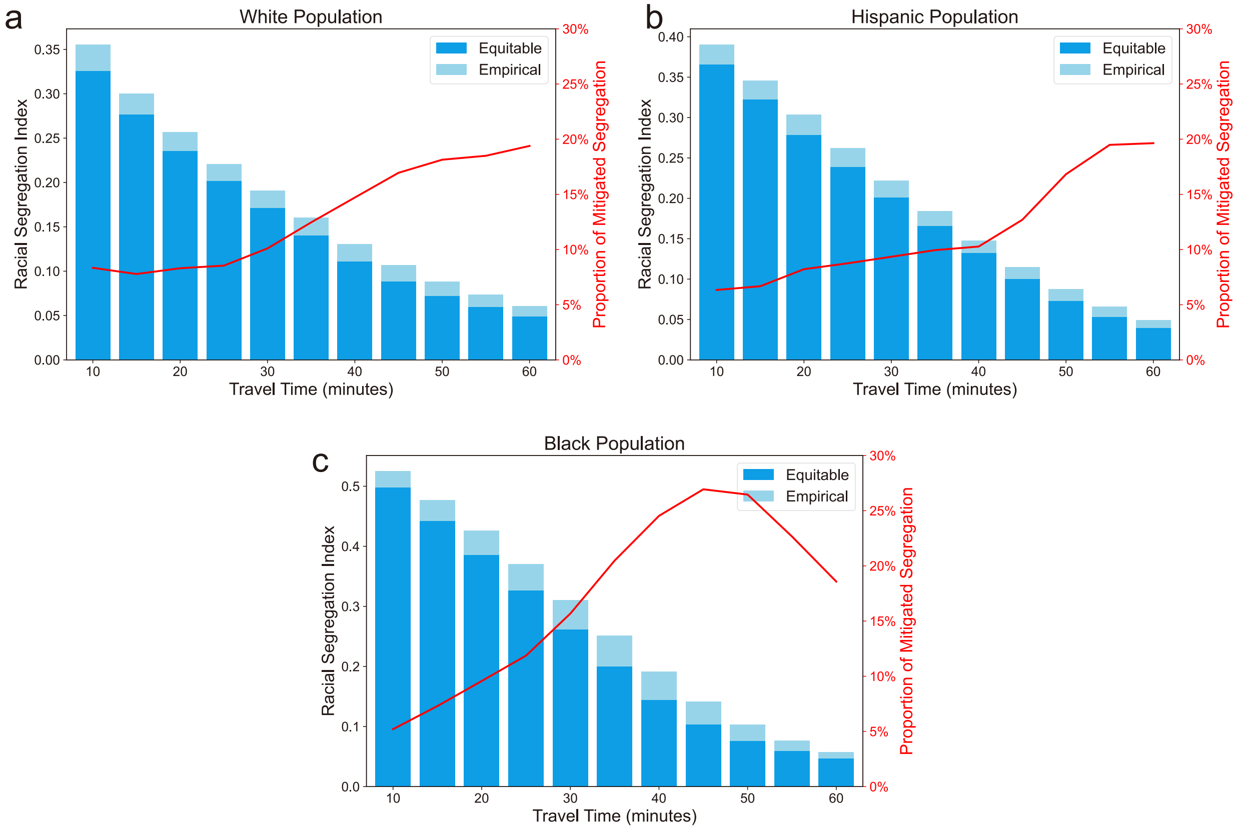

Figure S8. The average experienced segregation index S under empirical and equitable transit scenarios of white (a), Hispanic (b), and black(c) populations. Red lines denote the proportion of experienced segregation that can be mitigated by equitable transit networks, which is the difference in segregation indices divided by segregation index under the empirical scenario.

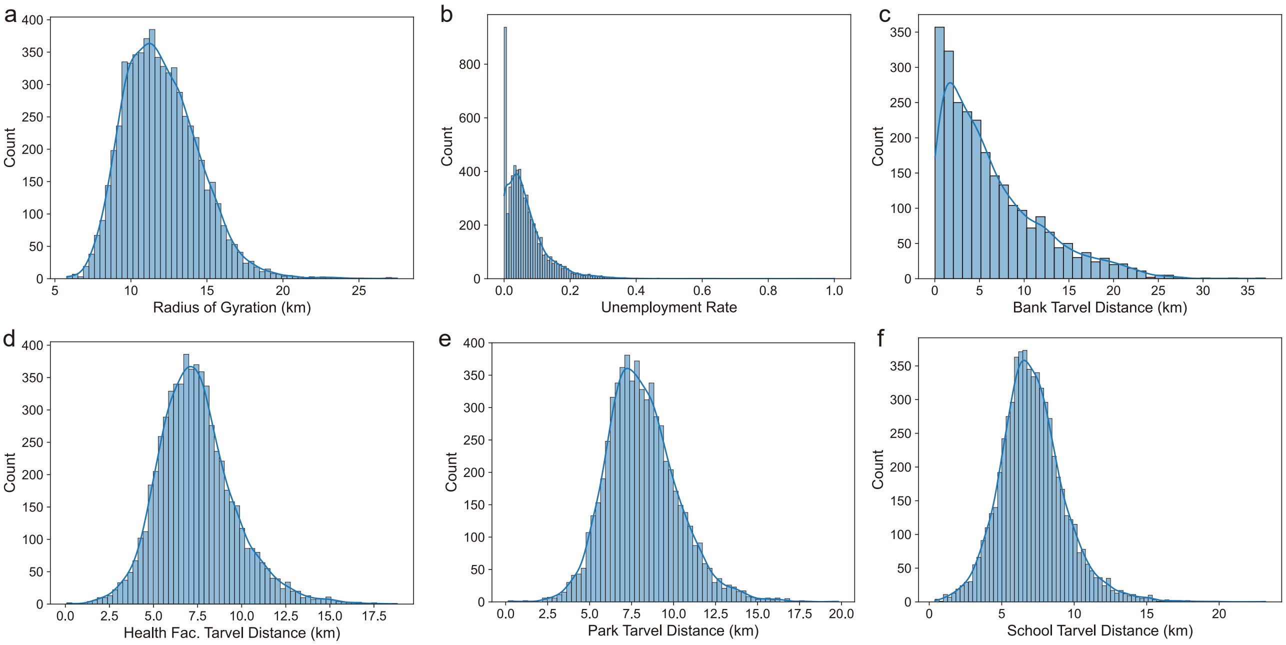

Figure S9. Distributions of block group real-world behaviors. (a) radius of gyration, (b) unemployment rate, average travel distance to (c) banks, (d) healthcare facilities, (e) parks, (f) schools.

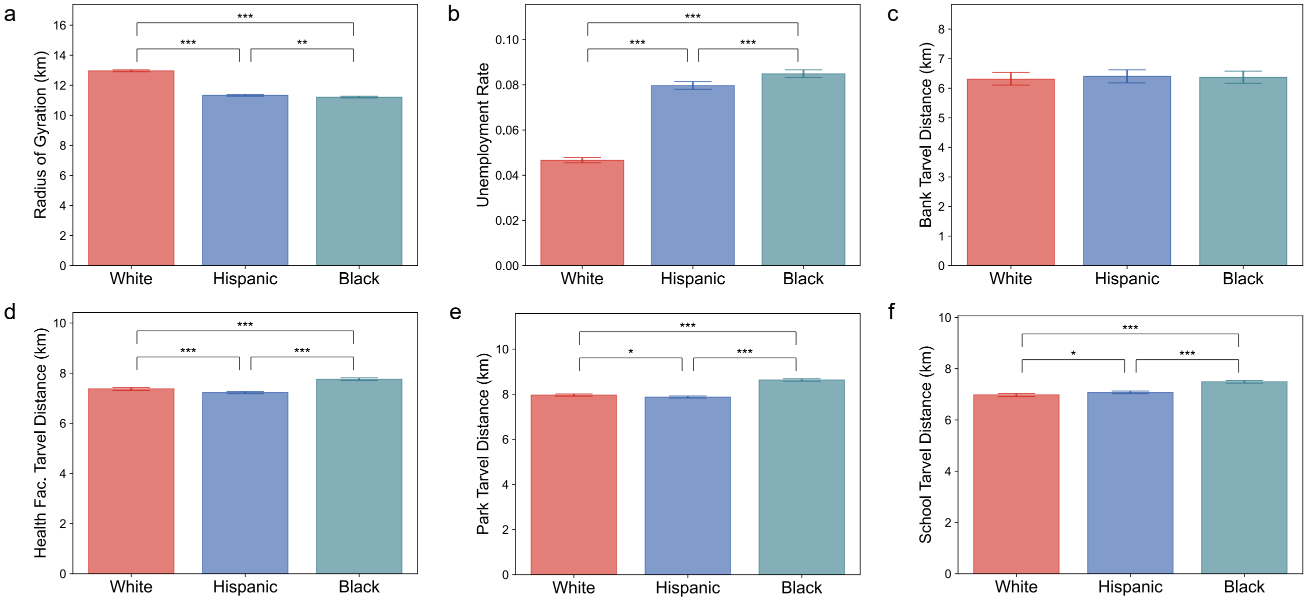

Figure S10. Discrepancies in real-world behaviors among block groups. The radius of gyration(a), unemployment rate(b), average travel distance to banks(c), healthcare facilities(d), parks(e), schools(f), and corresponding 95% confidence intervals in white, Hispanic, and black populations. Significance level: *: p<0.05, **: p<0.01, ***: p<0.001.

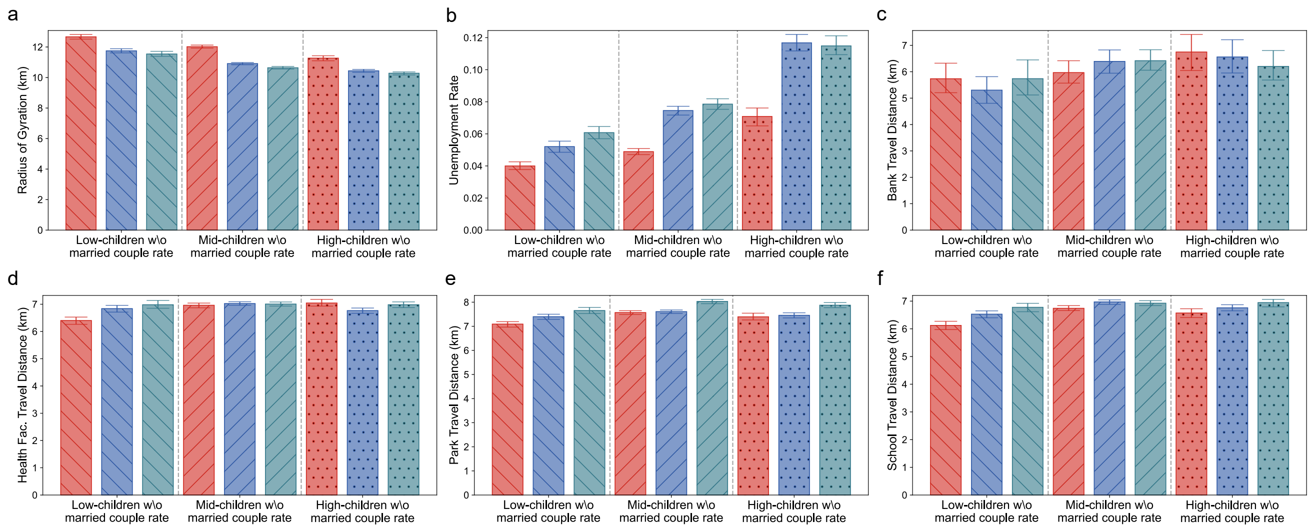

Figure S11. Discrepancies in real-world behaviors among block groups under different family structures. The radius of gyration(a), unemployment rate(b), average travel distance to banks(c), healthcare facilities(d), parks(e), schools(f), and corresponding 95% confidence intervals in white, Hispanic, and black populations under different levels of children living in non-married-couple family rates.

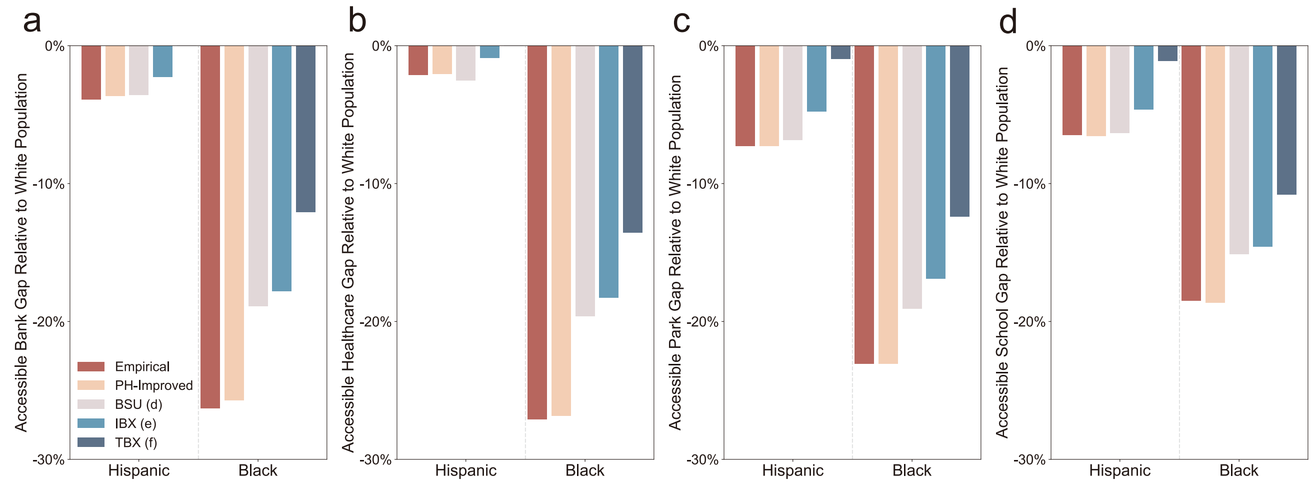

Figure S12. The relative accessibility gaps of Hispanic and black population under empirical and four simulation scenarios. (a) accessible banks. (b) accessible healthcare facilities. (c) accessible parks. (d) accessible schools.

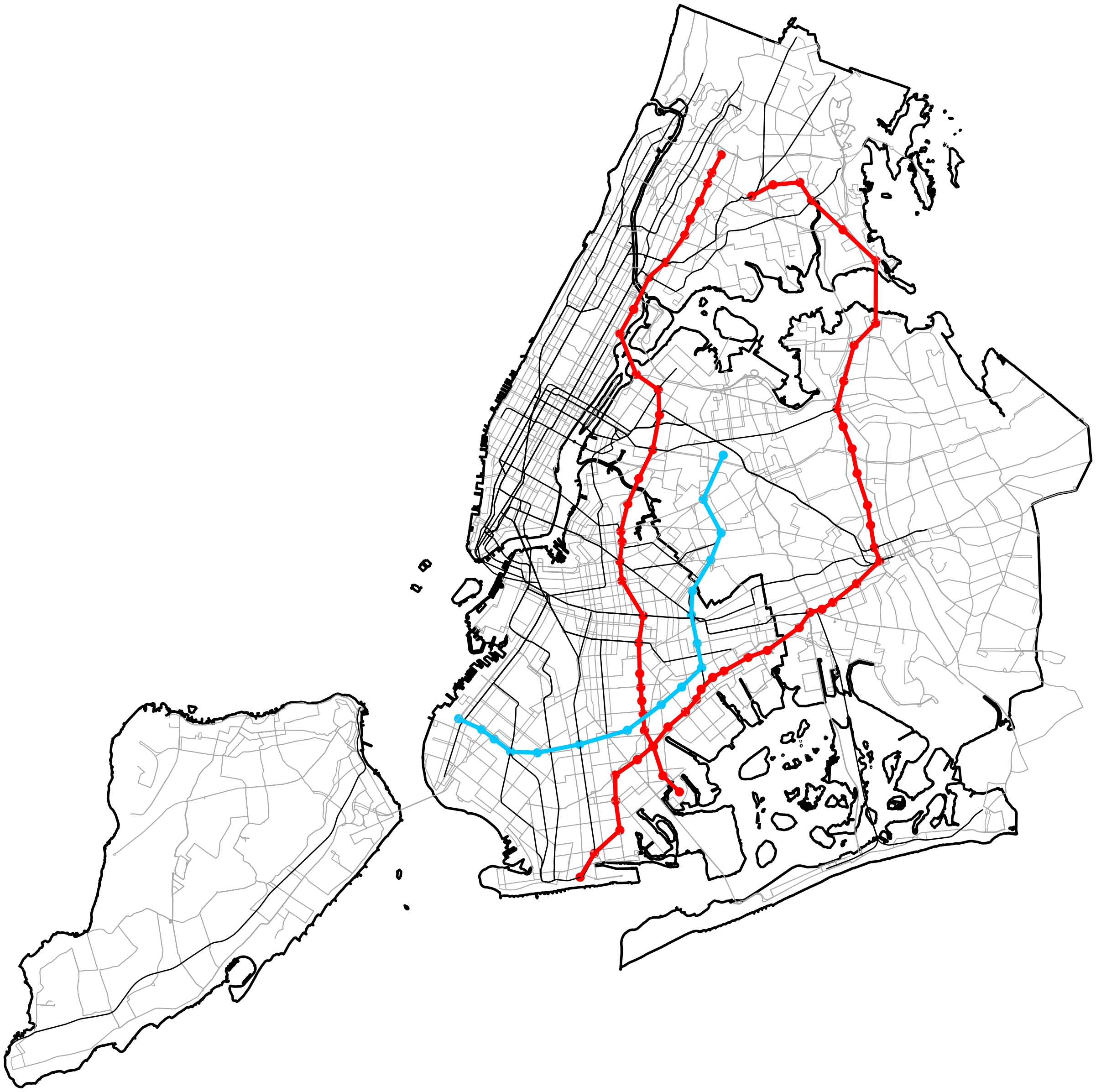

Figure S13. The spatial distribution of transit routes in five boroughs. Existing bus and subway routes are depicted as gray and black lines, respectively. The proposed IBX and TBX stations and routes are depicted as blue and red dots/lines.

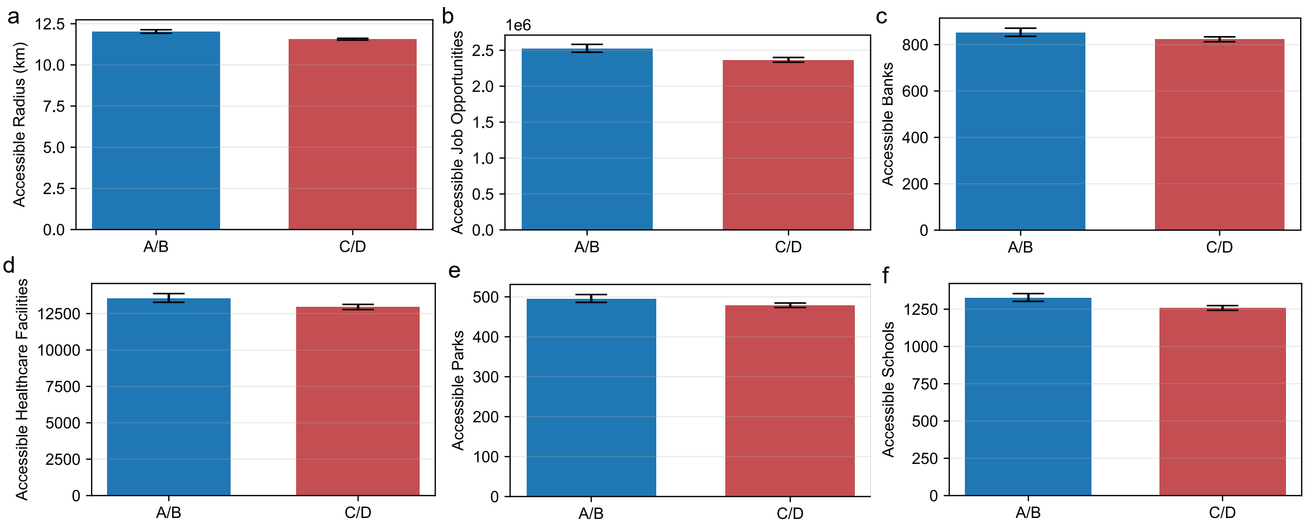

Figure S14. Population-weighted averages of accessibility metrics among block groups of different HOLC grades. Whiskers represent the corresponding 95% confidence intervals.

Supplementary Tables

Demograhpics	All block groups (6,290)			
	Min	Median	Mean	Max
population	122	1,244	1,358	9,963
household	0	452	511	4,970
non-Hispanic White ratio	0.0%	24.6%	33.6%	100%
non-Hispanic Black ratio	0.0%	6.8%	21.7%	100%
Hispanic ratio	0.0%	18.9%	27.9%	100%
annual median income ($)	8,493	68,162	75,263	250,000
poverty rate	0.0%	13.1%	17.4%	100.0%
unemployment ratio	0.0%	5.0%	6.5%	63.5%
public transit usage	0.0%	56.8%	55.0%	100.0%
household car ownership	0.0%	46.1%	49.1%	100.0%
foreign born rate	0.0%	35.4%	36.5%	95.8%
housing value (k$)	10.0	632.2	735.0	2,000.0

Table S1. Statistical characteristics of block group demographics.

Demograhpics	Public housing block groups (507)				Non-public housing block groups (5,783)			
	Min	Median	Mean	Max	Min	Median	Mean	Max
population	155	1,402	1,537	4,920	122	1,234	1,343	9,963
household	20	528	585	2,486	0	448	505	4,970
non-Hispanic White ratio	0.0%	14.7%	7.5%	86.3%	0.0%	29.8%	35.9%	100.0%
non-Hispanic Black ratio	0.0%	43.6%	45.3%	100.0%	0.0%	5.1%	19.6%	100.0%
Hispanic ratio	0.0%	38.3%	38.6%	96.2%	0.0%	18.0%	26.9%	100.0%
annual median income ($)	8,661	27,641	41,001	210,592	8,493	70,417	78,173	250,000
poverty rate	0.0%	34.0%	33.5%	82.9%	0.0%	12.2%	15.9%	100.0%
unemployment ratio	0.0%	9.9%	12.6%	63.5%	0.0%	4.8%	6.0%	46.6%
public transit usage	0.0%	65.4%	63.2%	100.0%	0.0%	56.2%	54.3%	100.0%
household car ownership	0.0%	23.1%	32.3%	100.0%	0.0%	47.8%	50.6%	100.0%
foreign born rate	4.4%	26.4%	28.5%	79.5%	0.0%	36.5%	37.2%	95.8%
housing value (k$)	61.5	459.3	565.6	2,000.0	10.0	641.5	741.9	2,000.0

Table S2. Statistical characteristics of public housing and non-public housing block group demographics.

Transit Agency	Route Type(s)	Number of Routes
NYC Bus Company	Bus	90
NYC Subway	Subway / Metro; Rail / Train	30
Long Island Rail Road	Rail / Train	12
Metro-North Railroad	Rail / Train	6
MTA Bronx	Shuttle Bus; Bus	281
MTA Brooklyn	Shuttle Bus; Bus	
MTA Manhattan	Shuttle Bus; Bus	
MTA Queens	Shuttle Bus; Bus	
MTA Staten Islands	Shuttle Bus; Bus	
NYC Ferry	Ferry; Bus	9
Staten Island Ferry	Ferry	1

Table S3. Collection of New York City GTFS datasets in 2022.

**Category**	**NAICS Codes**
Banks	(522110)
Healthcare Facilities	(621111, 621112, 621210, 621310, 621320, 621330, 621340, 621399, 621410, 621492, 621493, 621498, 621511, 621512, 621610, 621910, 621991, 621999, 622110, 622210, 622310, 623110, 623311, 623312, 624110, 624120, 624190, 624210, 624221, 624410)
Parks	(712190)
Schools	(611110, 611210, 611310)

Table S4. NAICS (North American Industry Classification System) codes included in each category.

Month	No. of mobile devices	No. of visits
March 2019	730,974	21,405,396
April 2019	745,775	22,034,553
May 2019	743,477	22,564,475

Table S5. Basic statistics of the SafeGraph mobility dataset.

Dependent variable	Mediation variable	c	a	c'	b	Mediation proportion
Unemployment rate	Accessible jobs	0.0127	-2.4794×10^{5}	0.0115	-4.8972×10^{-9}	9.56%
Health travel distance	Accessible healthcare	0.4657	-1.3277×10^{3}	0.3006	-1.2436×10^{-4}	35.46%
Park travel distance	Accessible parks	0.5539	-26.2221	0.4446	-4.1666×10^{-2}	19.67%
School travel distance	Accessible schools	0.3852	-38.1208	0.3189	-1.7395×10^{-3}	17.17%

Table S6. Estimated regression coefficients in mediation analysis and corresponding mediation proportion of accessibility differences on behavioral discrepancies.
